# Mendel,MD: A user-friendly open-source web tool for analyzing WES and WGS in the diagnosis of patients with Mendelian disorders

**DOI:** 10.1371/journal.pcbi.1005520

**Published:** 2017-06-08

**Authors:** Raony G. C. C. L. Cardenas, Natália D. Linhares, Raquel L. Ferreira, Sérgio D. J. Pena

**Affiliations:** 1 Laboratório de Genômica Clínica, Faculdade de Medicina da UFMG, Universidade Federal de Minas Gerais, Belo Horizonte, Brazil; 2 Departamento de Bioquímica e Imunologia, Instituto de Ciências de Biológicas, Universidade Federal de Minas Gerais, Belo Horizonte, Brazil; 3 GENE–Núcleo de Genética Médica, Belo Horizonte, Brazil; University of Canterbury, NEW ZEALAND

## Abstract

Whole exome and whole genome sequencing have both become widely adopted methods for investigating and diagnosing human Mendelian disorders. As pangenomic agnostic tests, they are capable of more accurate and agile diagnosis compared to traditional sequencing methods. This article describes new software called Mendel,MD, which combines multiple types of filter options and makes use of regularly updated databases to facilitate exome and genome annotation, the filtering process and the selection of candidate genes and variants for experimental validation and possible diagnosis. This tool offers a user-friendly interface, and leads clinicians through simple steps by limiting the number of candidates to achieve a final diagnosis of a medical genetics case. A useful innovation is the “1-click” method, which enables listing all the relevant variants in genes present at OMIM for perusal by clinicians. Mendel,MD was experimentally validated using clinical cases from the literature and was tested by students at the Universidade Federal de Minas Gerais, at GENE–Núcleo de Genética Médica in Brazil and at the Children’s University Hospital in Dublin, Ireland. We show in this article how it can simplify and increase the speed of identifying the culprit mutation in each of the clinical cases that were received for further investigation. Mendel,MD proved to be a reliable web-based tool, being open-source and time efficient for identifying the culprit mutation in different clinical cases of patients with Mendelian Disorders. It is also freely accessible for academic users on the following URL: https://mendelmd.org.

This is a *PLOS Computational Biology* Software paper.

## Introduction

Whole exome sequencing (WES) and whole genome sequencing (WGS) have revolutionized clinical genetics through the discovery of new genes, the characterization of new genetic diseases, and the description of new phenotypic features in previously known disorders [1–3]. The efficiency of WES and WGS in unraveling Mendelian Disorders originates from the collective characterization of genes in a pangenomic, agnostic, non-targeted fashion. Variants that are present in all expressed human genes are analyzed in parallel, using multiple filter options while searching for the “culprit” variant in each clinical case. Such a process depends on software that ideally should be easy to use by clinicians, who sometimes have limited knowledge of computing. Thus, in the best of all possible worlds, computer tools for genomic analysis should be simple, intuitive and user-friendly.

Currently there are already a few commercial tools that attempt to address this problem such as Variant Analysis from Ingenuity[4], VarSeq from Golden Helix[5] and Sequence Miner from Wuxi NextCode[6]. Also, there are a few open source tools such as GEMINI[7], seqr[8], VCF-Miner[9], BiERapp[10], BrowseVCF[11] that also aim to provide a Graphical User Interface to simplify the analysis of the genetic information of a patient. On Table 1 we provide a feature grid comparing Mendel,MD with the other tools available.

**Table 1:** Feature grid of tools for human genome annotation and analysis. This table shows the comparison of multiple tools and platforms that can be used for doing variant annotation, prioritization and clinical genome analysis.

Features	Mendel,MD	GEMINI	Ingenuity Variant Analysis	VarSeq	Sequence Miner	seqr	VCFMiner	BiERapp	Browse-VCF
**Open Source**	✓	✓	✘	✘	✘	✓	✓	✓	✓
**Easy to use**	✓	✘	✓	✓	✘	**Not public**	✘	✘	✘
**Web Interface**	✓	**Partially**	✓	✓	✓	**Not public**	✓	✓	✓
**Public Server** **Available**	✓	✘	✘	✘	✘	✘	✘	✓	✘
**Example Datasets with Mendelian Disorders**	✓	✘	✘	✘	✘	**Not public**	✘	✘	✘
**Multi Sample Comparison**	✓	✓	✓	✓	✓	✓	✓	✓	✓
**Annotation**	✓	✓	✓	✓	✓	✓	✓	✓	✓
**Filtering**	✓	✓	✓	✓	✓	✓	✓	✓	✓

Mendel,MD uploads a VCF file, annotates it, inserts it to a database and finally filters it. For this process, it makes use of a simple web interface that can be freely accessed from any computer, tablet or smartphone with any Internet browser.

The goal of Mendel,MD is not to provide a single candidate gene, but rather a limited list of good candidates that can always be manually investigated by researchers and doctors using their research and clinical skills. One innovative strategy we tried to develop was the option for a “1-Click” automatic search that makes use of minimal pre-set of filter options and thresholds to produce a list of candidate variants in genes included at the Online Mendelian Inheritance in Man (OMIM) [12] and at the Clinical Genomic Database (CGD) [13]. The user can also, if they wish, add extra options of filters for different modes of inheritance, for chromosomal positions, variant effects, functional classes, variant frequencies and pathogenicity scores among other options.

## Design and Implementation

Mendel,MD was developed to be compatible with Python 2.7 and 3.x. We developed the web interface using the Django web-framework[14]. We used different methods, tools and sources of information to generate at the end of the process a fully annotated VCF file [15] with all the necessary information for the selection of good candidate variants and genes that could be responsible for causing the disease in multiple different clinical cases.

This data is inserted into a PostgreSQL database in order to facilitate the filtering of each patient’s variants through a web browser (see an example of this annotated VCF file in S1 Data).

**Fig 1:**
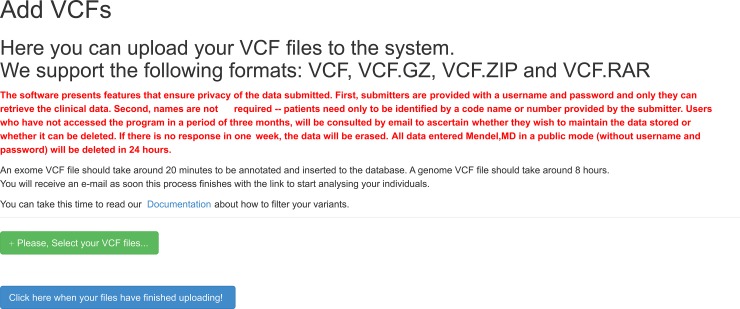
Upload System. This figure shows the interface for submission of VCF files in the system using a library called JQuery File-Upload. As soon the user selects the VCF files from his computer, the upload starts automatically and there is an estimated time showing that it is updated constantly until the completion of the upload.

The first thing we developed was the upload system using a JavaScript library called JQuery File-Upload[16] which enabled the ability of a user to simply drag-and-drop VCF files from his desktop into the browser or to select multiple VCF files and upload all at once to Mendel,MD. The current system accepts the following formats for upload:.VCF,VCF.GZ, VCF.ZIP and VCF.RAR. In Fig 1 we present the web interface of the upload system.

### Diseases

In order to aggregate information about Mendelian Disorders into our database we used two main sources of information: the Online Mendelian Inheritance in Man (OMIM) [12] and the Clinical Genomic Database (CGD) [13]. The list of genes is always compared live for each filter analysis search to allow, for example, the investigation of variants only in genes previously known to be associated with Mendelian Disorders. In the “Disease” section of Mendel,MD, it is possible to search for diseases by their names or by the gene symbols associated with them (Ex. ‘*Mitochondrial depletion syndrome 5*’ or ‘*SUCLA2’*) and quickly retrieve a list of genes and diseases associated with every term. From the results of this search, it is possible to select a list of genes and search for variants only in the selected genes screening all the individuals present in our database.

### Genes

We added to the database the official list of gene symbols and descriptions from the HUGO Gene Nomenclature Committee (HGNC) website, which currently has 19,006 protein-coding genes. In the “Genes” section of our tool it is possible to search for gene symbols and gene names, (Ex. *ASS1P1* or *argininosuccinate*) and select from the list of genes to visualize variants in all the individuals present in our database.

### Annotation framework

**Fig 2:**
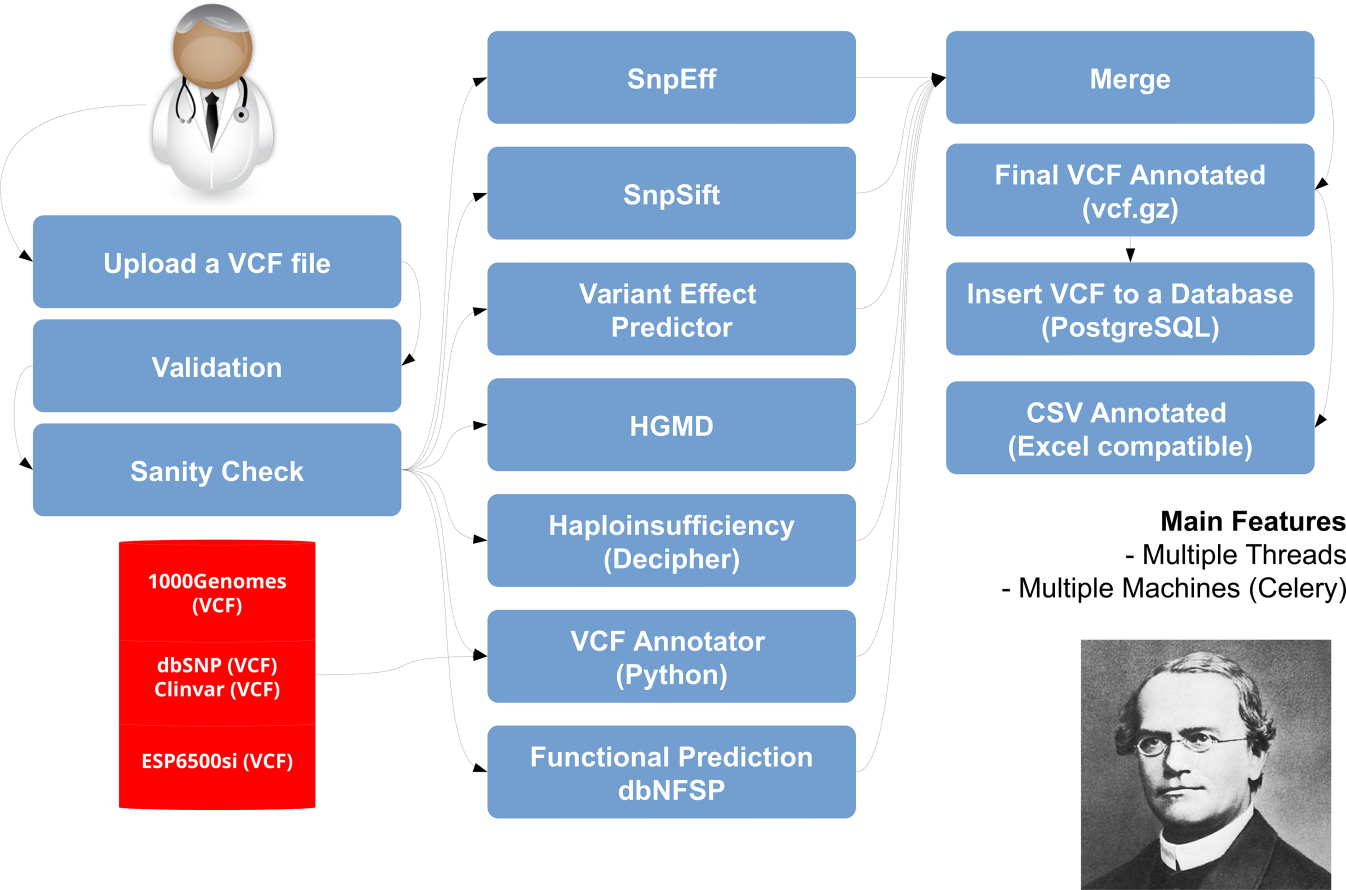
Pynnotator annotation framework. This figure shows the processes we perform on each VCF file after a user uploads it to the system. First we perform a vcf-validation and a sanity check to make sure the file is ready to be annotated. After it, we send each checked VCF file to be annotated in parallel by SNPEFF, SNPSIFT, VEP, Decipher, and two other python scripts we developed in-house. The first one is capable of using other VCF files to annotate a VCF file so we use the VCFs from 1000Genomes, dbSNP and NHLBI GO Exome Sequencing Project to annotate a VCF and the second python script uses data from dbNSFP to add functional annotation and prediction scores to our annotation. After merging the results of all tasks we compress the file and prepare it to be inserted to the database.

We used a Distributed Task Queue system called Celery [17] to annotate multiple VCFs in parallel. This tool enables the possibility of scaling the annotation of VCF files using a cluster of computers in order to speed up this process and also to execute it faster in bigger machines. We used 4 queues to annotate VCFs, parse the results and insert the final results into our database. In Fig 2, we present the annotation framework that we called pynnotator[18], which was developed together with Mendel,MD. Next we describe in more detail how this annotation framework works.

After a user submits a VCF file, the first step our framework performs is the validation of each file using a method called “vcf-validator” from VCFtools [15]. After doing this validation, we execute a python script called “sanity-check” to prepare the VCF to be annotated by Mendel,MD. This script searches and removes lines of the VCF files that contain the genotype “0/0”, removes the “chr” letters from the beginning of each chromosome name, sorts all the variants of the VCF by chromosome name and position, and finally it removes the EFF tag of any prior annotation that was done with SNPEFF in the past. Another tool that provides a similar functionality is VCFAnno[19].

After validating and checking each file, we make use of the “threading” module library of Python to execute the following tools in parallel: SNPEFF[20] and SNPSIFT[21], Variant Effect Predictor (VEP)[22] and “vcf-annotate” from VCFtools [23]. Following this, we use a python script called “vcf-annotator.py”, which is an important step of our annotation since it is a generic form used to annotate any VCF file using multiple VCF files as a reference. This script itself also uses multiple threads in order to make this particular part of the annotation more efficient.

We use the following projects and databases as reference for the annotation task: 1000 Genomes Project [24], dbSNP and Clinvar [25], Exome Sequencing Project (ESP) [26] and dbNFSP [27]. These files were downloaded and stored using the BGZIP format and were indexed using tabix [28] which helped reduce the amount of space required to perform our annotation (30GB) while keeping the files indexed and enabling fast information retrieval based on the genome coordinates. The library pysam [29] was used for interfacing with tabix to access the required information.

Finally we used two VCF files with information from the public HGMD mutations (downloaded from Ensembl) and the Haploinsufficiency Index of some genes as calculated by Huang et al [30]. At the end of our annotation process, we merge all the output of the tools used into a final VCF file containing hundreds of annotated fields added to the column INFO at every line that was present in the original file. This file contains the annotation for various scores of pathogenicity such as SIFT[31], PolyPhen-2 [32], VEST [33] and CADD [34], and these scores are very important for evaluating the pathogenicity of each variant and can help select good candidates for each clinical case.

In S1 Data we present an example of a VCF file annotated by Mendel,MD. We noticed earlier in this project that the task of re-annotating each VCF file would need to be repeated many times in order to keep this information updated. To address this challenge we created a page called “Dashboard” where a user with administration privileges can quickly select individuals and send them to be re-annotated every time new datasets and tools would be provided from upstream. We developed this process in a way that new tools and datasets could easily be integrated into it, so that changes could constantly be made with the goal of improving the quality of the analysis.

After the annotation was finished we inserted each annotated VCF into an SQL database developed using PostgreSQL in order to store, index, and quickly retrieve this information. To take care of filtering variants from multiple individuals we developed a method called “Filter Analysis”. Next we describe how this method is useful for excluding variants according to filter options pre-defined by the user.

**Fig 3:**
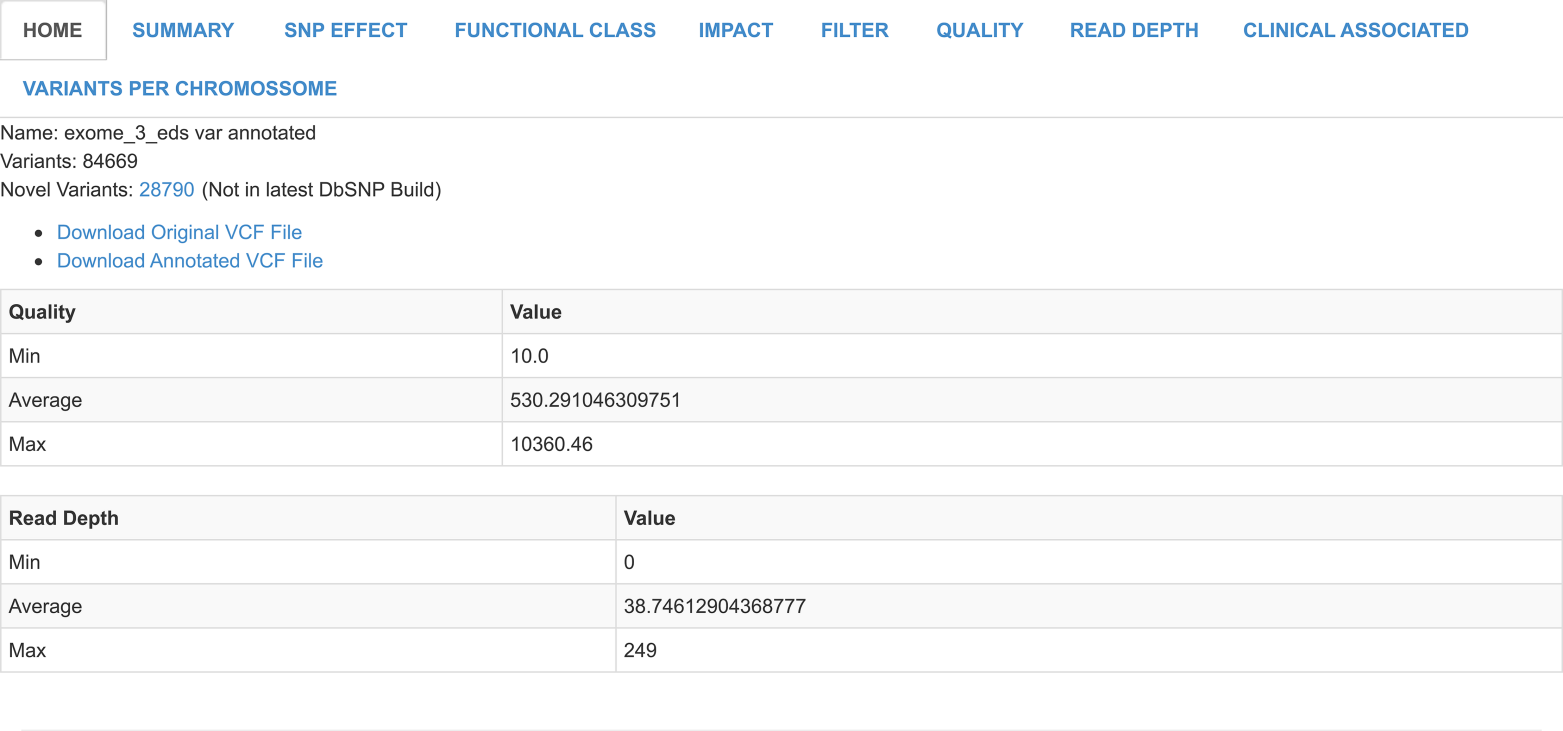
VCF summary. Here you can see some metrics for each VCF file such as the total number of variants and the number of novel variants.

In Fig 3 we show a summary for a VCF file with metrics about the read depth, quality score and total number of variants in order to help define thresholds for the next section implemented, which is called Filter Analysis.

### Filter analysis

To implement the filtering of the VCF data we made extensive use of the Django Object-relational mapping (ORM) which is capable of translating python code directly into SQL queries, thus facilitating the process of building complex queries that can be combined with the goal of reducing the number of candidate variants and genes for each different clinical case.

**Fig 4:**
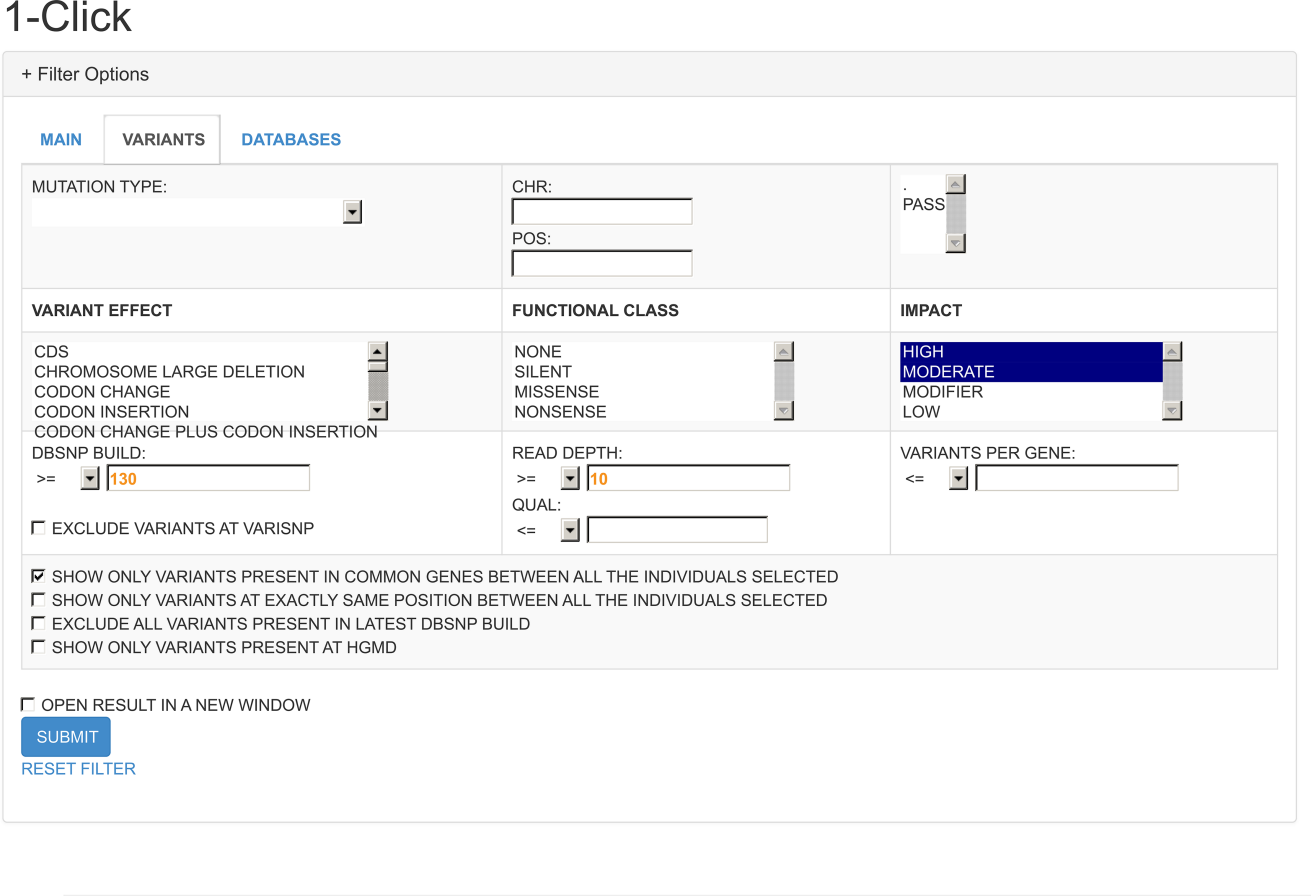
Filter analysis. Using this method, a user can quickly define multiple options in order to exclude variants according to different criteria. This enables you to quickly repeat the filtering step using different options to adjust your analysis according to your preferences.

In Fig 4 we show the interface that was developed for filtering these variants based on the fields from the VCF that were annotated and inserted into the database. With these options a user can exclude variants based on certain fields such as the type of mutation (e.g. homozygous or heterozygous), the impact of mutation according to SNPEFF (Ex. high, moderate, modifier or low), and even the frequency of the mutation according to the databases 1000 Genomes, dbSNP and Exome Sequencing Project.

It is also possible to search for variants only in genes previously known to be associated with Mendelian disorders. We implemented autocomplete fields where the user can type a word and quickly search and retrieve a list with the possible options of diseases with this term to add to their search. This feature can speed up the process of increasing the options and also it allows the user to search for variants only in genes associated with specific diseases. We made this part of the analysis user-friendly so that it could be easily performed by doctors and researchers. This feature can greatly hasten the identification of good candidate variants for experimental validation. In the results section of this search, the user can see a list of genes that are already known to be associated with Mendelian Disorders in the OMIM and the Clinical Genomics Database and decide to focus only on variants present in these genes. This is a good strategy that can help markedly reduce the number of candidate variants that may cause a Mendelian Disorder.

### 1-Click

We created this method by defining standard values for the fields that were available in the previous method Filter Analysis. The suggested default values for filtering are the following: Exclude all variants that were included in the dbSNP build 129 (this was the last dbSNP build that did not contain pathogenic SNVs) or lower, exclude all variants with a read-depth value lower than 10, show only variants with a HIGH or MODERATE impact as classified by SNPEFF, show only variants present in common genes between all selected individuals and finally exclude variants with frequency lower than 1% in the following databases: 1000Genomes, dbSNP and ESP6500. These simple rules will already produce a list of genes and variants that should be investigated manually.

**Fig 5:**
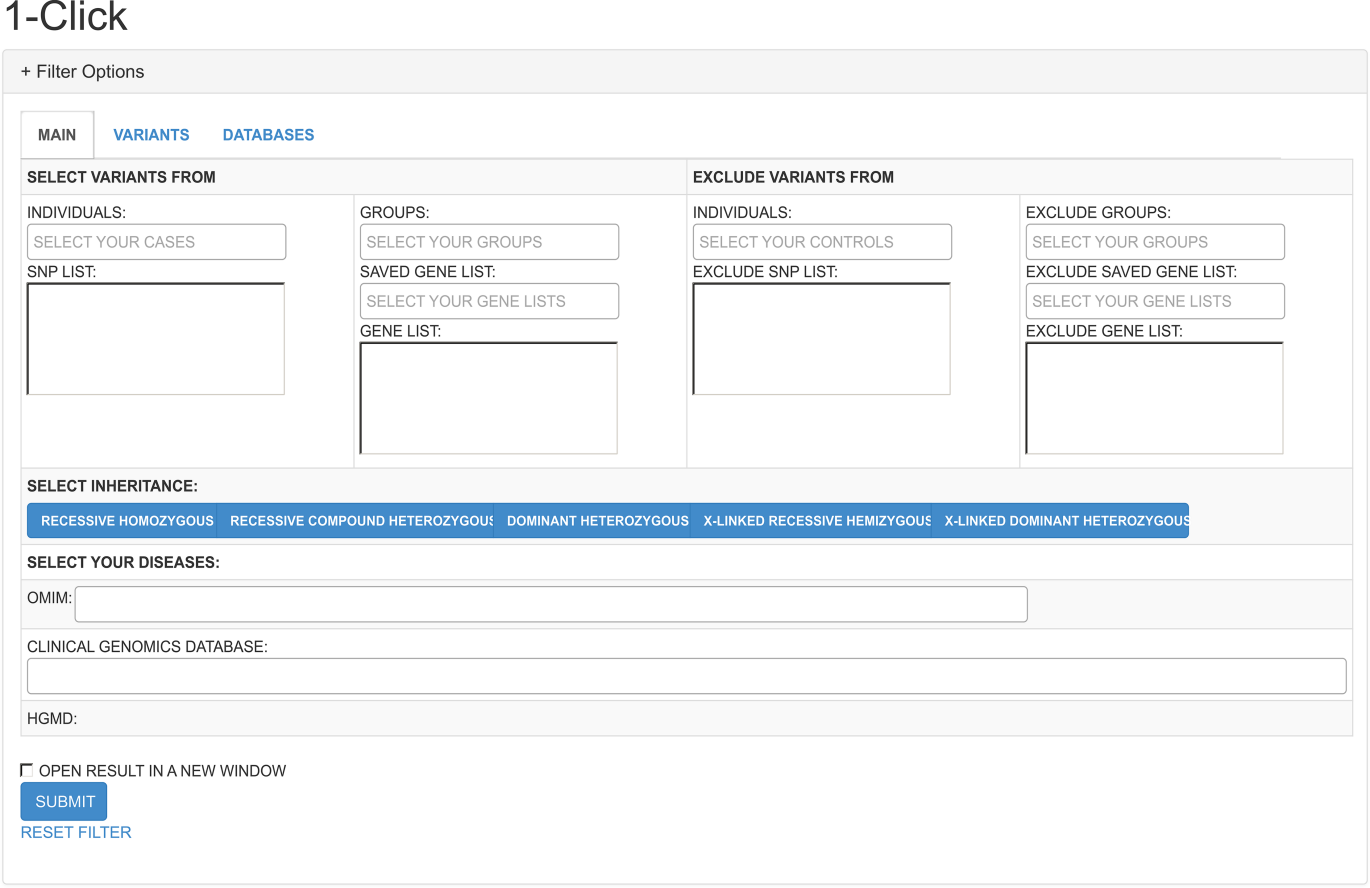
1-Click. This method was built to allow the use of pre-defined filters and was integrated with different models of inheritance and phenotypes available in our database.

In Fig 5 we present the interface we called 1-Click and where it is possible to see the available options such as select for different modes of inheritance and specific diseases that are available. We chose not to add any scores of pathogenicity as a standard option for this method so not to exclude any variants from the initial list that could be wrongly classified by one of these scores. Here we decided to use a more conservative approach and let users decide whether or not they want to use pathogenicity scores to filter their candidate variants.

**Fig 6:**
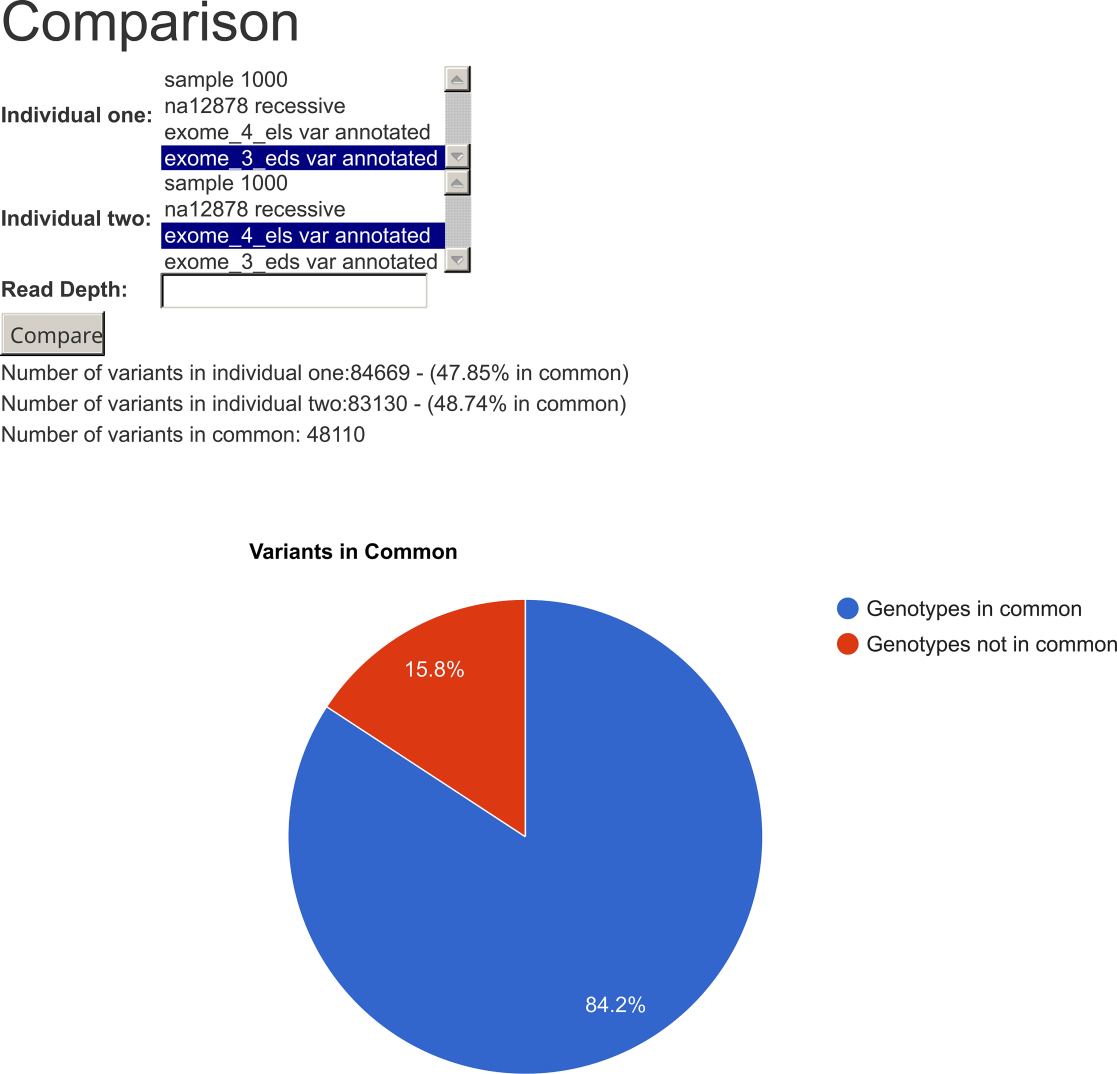
VCF comparison. This method searches for variants at common positions in samples (Ec. Two VCF files), and compares the genotypes in all these common positions. Here we show that two siblings had 84.2% of genotypes in common in the positions their VCF have in common.

In Fig 6 we present a method called “VCF comparison,” which can be used to perform a quick comparison between two VCF files. Here we compared the genotypes of two siblings and the result shows that they have 48,110 positions in common and also 84.2% of the genotypes at these positions are the same. This method can also be used to compare VCF files from the same individual but generated using different parameters or techniques. For instance, it is ideal to identify the somatic mutation of malignant tumors, by comparing the cancer exome with the germinative exome of the same individual.

## Results

We empirically tested Mendel,MD in real life situations for efficiency and ease of use-cases from the literature, real life clinical exome analysis and analyses by graduate students. We will describe these empirical tests in the following paragraphs.

### Tests based on cases from the literature

We first used data of successful validated previous cases already published in the literature in recent years. We sent e-mails to the authors of these studies asking for their patients’ data to use while performing the validation of Mendel,MD. We received a total of 19 exome VCF files from 11 different clinical cases for this validation.

In S1 Table we present a list with the clinical cases and exomes that we received. We also had the information about the model of inheritance for each clinical case. In S2 Table we present the number of variants for each exome and some statistics such as the minimum, maximum and mean of coverage and quality for each individual.

We wanted to test if physicians and researchers would be able to use Mendel,MD to identify candidate genes and mutations for each clinical case. In order to make this validation more real, we removed the name of the Mendelian Disorder and asked a medical doctor to create a list of symptoms for each clinical case. We prepared a spreadsheet with a list of symptoms and the inheritance model for each clinical case. We made these data available to members of our laboratory to ascertain whether they would be able to identify the right genes and variants for each clinical case. Using Mendel,MD, all of them successfully independently identified the correct gene and variant for all the clinical cases.

In S1 Text we describe how the analysis of each clinical case was done. In all cases we used the standard method called 1-Click, selecting the inheritance model reported and adjusting the read depth in some cases according to the average of coverage of the exomes provided.

### Tests of efficiency based on diagnostic use

Clinical exome sequencing was performed on 57 patients with undiagnosed, suspected genetic conditions at the GENE–Núcleo de Genética Médica in Belo Horizonte, Brazil. Clinical exome sequencing was conducted only in the proband. All variants considered pathogenic were confirmed by Sanger sequencing of the patient and the family. The parents were also studied to establish the phase of compound heterozygous variants and to permit the identification of *de novo* heterozygous variants. All cases were studied with the 1-Click route of Mendel,MD, followed by intensive clinical scrutiny of the list produced of potentially pathogenic variants. Using this methodology, a definitive diagnosis could be reached in 29 of the 57 cases (51%).

Also, 42 children with early onset epileptic encephalopathy were submitted to diagnostic WES and analyzed exclusively with Mendel,MD at the Children’s University Hospital, Dublin, Ireland. “Disease causing” variants were identified in 26% of the patients [27]. Additionally one novel gene (NAPB) associated with early-onset epileptic encephalopathy was identified in this study in a 6-year-old girl with a homozygous nonsense variant at cDNA.565C>A (chr20:23370665) [35].

### Tests of ease-of-use based on experiences of graduate students

In the second semester of 2014, SDJP administered a course in human molecular biology to seven students from the Graduate Course in Biochemistry and Immunology of the Universidade Federal de Minas Gerais.

As one of the many activities of the course each student was provided with the WES VCF file of one of the patients diagnosed at GENE–Núcleo de Genética Médica (see previous subsection), accompanied by a succinct two-line anonymous clinical summary (list provided in S1 Table). The students, none of whom had any experience in exome analysis, had to complete the assignment in one week.

They did the exome analysis exclusively using Mendel,MD. Six of the seven students were successful in identifying the correct “culprit” variation. Following the end of the course the students were given an optative multiple-choice questionnaire about the use of Mendel,MD with two questions: (1) what grade do you attribute to your experience with Mendel,MD (A 0–20, B 30–40, C 40–60, D 70–80, E 90–100)? (2) how do you evaluate Mendel,MD compared with other software that you have used? (A Very easy, B Easy, C Average, D Difficult, E Very difficult).

Four students replied: three of them rated Mendel,MD as 90–100 and the fourth as 70–80; three of the graduate students rated the program Easy and the other Average. Although small in scale this simple study was considered meaningful in showing that Mendel,MD is effective, easy and user-friendly software.

### Example datasets and educational aspects

We used a public VCF from one individual of the 1000 Genomes Project (NA12878) and prepared a tutorial with 4 different VCFs each one with a different Mendelian Disorder. We added the following types of inheritance: Autosomal Recessive–Homozygous, Autosomal Recessive—Compound Heterozygous, Autosomal Dominant–Heterozygous and Dominant X-linked–Hemizygous. This VCFs can be used to test our tool and train users searching for the culprit of each different clinical case.

## Availability and future directions

Mendel,MD is an open-source project under the 3-clause BSD License. In order to execute Mendel,MD you will need a computer with at least 4GB of RAM and at least 60GB of hard disk space. We offer the full source code of our tool on Github with the docker instructions. It can be downloaded and installed in any UNIX machine (preferably Ubuntu LTS) using the automated installation script provided or on any computer using Linux Docker.

Source code: https://www.github.com/raonyguimaraes/mendelmd.

We tested the performance of this tool by annotating and entering hundreds of exomes into our database. We used a tool called PgTune [36] to increase the performance of our PostgreSQL database according to our hardware specifications.

## Supporting information

S1 Text Supplementary material. Description of how each sample was analyzed using Mendel,MD.
(DOCX)

S1 Table Description of the analysis of each clinical case received. Information about the 11 different clinical cases received, file types, types of inheritance and the small description of the symptoms generated for each clinical case.
(XLSX)

S2 Table QC metrics about each VCF file received for validation. Number of variants and the mean of coverage for each sample received.
(XLSX)

S1 Code Mendel,MD software source-code. Last version of the source-code of Mendel,MD.
(ZIP)

S2 Code Pynnotator VCF annotation framework source-code. Last version of the source code of Pynnotator.
(ZIP)

S1 Data Example of a VCF file annotated by Pynnotator. This is an example VCF file to show all genomic annotations that are added at the INFO column when using the Pynnotator annotation framework.
(ZIP)
