# Supplementary material for: Mendel,MD: A user-friendly open-source web tool for analyzing WES and WGS in the diagnosis of patients with Mendelian disorders
Source: PLoS Comput Biol. 2017 Jun 8;13(6):e1005520. doi: 10.1371/journal.pcbi.1005520 (PMC5464533; doi:10.1371/journal.pcbi.1005520)
Supplement: S1 Text — Description of how each sample was analyzed using Mendel,MD. (DOCX) [file pcbi.1005520.s001.docx]

**Supplementary Information**

Case 1 was represented by an individual (DG00658) with a recessive Mendelian disorder, 86.445 variants and 63.25 of average of coverage. We used 1-Click with the default options and it returned 29 genes candidates from which 10 were already present in the OMIM database. We compared diseases associated with those genes and the symptoms described for this case: *Short Stature, Onychodysplasia, Facial Dysmorphism, and Hypotrichosis*, and finally we selected the gene *POC1A*[geneid:25886] as being the most likely candidate to cause the disease of this patient. The gene had the mutation *G>A* at the position *52183866* that was STOP GAINED, with no scores of pathogenicity such as sift or polyphen2, but with a very high CADD score of 7.57.

When solving case 2, we came up with 10 gene candidates, 4 of them being already mentioned at OMIM By comparing the list of genes with the list of symptoms we selected gene *MRPL44*[geneid:65080] as a most likely candidate for this specific clinical case. In this gene there was a homozygous mutation at position 2:224824538, that is *non-synonymous*, *MISSENSE* with a *SIFT Score* of 0 and a *Polyphen2* score of 0.95.

In case 3, we found 22 candidate genes, with 9 being already present at OMIM and we selected the gene *AARS*[geneid:16] as the most likely candidate to cause the disease of a patient. The gene had a homozygous mutation at the position 6:44272249, that was non-synonymous, missense, with sift score of 0 and a polyphen2 of 0.81.

Case 4 was represented by two individuals (nl1001037 and nl1000380). This time under the dominant model we found 63 genes from which 11 genes were already present at OMIM and we selected the gene *WDR35*[geneid:57539] as the most likely candidate to cause the disease of patients. The gene had 3 mutations that we found: one in an exome of the individual nl1001037 which was heterozygous, 2:20133230, non-synonymous, missense, with a sift score of 0 and a polyphen2 of 1.0. For the other individual (nl1000380) we identified two candidate mutations: one non-synonymous and one at a region of splicing.

In case 5, we found 25 genes present at OMIM, from which we selected the gene *MAX* [geneid:4149] that contained 3 different mutations.

In Case 6 we had four individuals to analyze: nl54126, nl48062, nl43664, nl40816. We used the dominant inheritance model which returned 19 gene candidates, from this list only four genes were already present at OMIM database and we selected gene: *SETPB1*[geneid:26040] as the most likely candidate to cause the disease based on the list of symptoms.

In Case 7 we had only one individual to analyze: DG1365. Using the recessive inheritance model we found 23 gene candidates, with 4 genes present at OMIM. Since none of those genes had seen to be associated with the list of symptoms, we reduced the minimum read depth to 5 and then we identified the gene *DOCK6*[geneid:57572] according to the list of symptoms for this clinical case. It was important to discover through this specific case that the standard values used for filter analysis might not always be optimal.

For cases 8 and 9 we did not have any symptoms listed, we only knew that the inheritance model was compound heterozygous. There was a problem with the scripts which converted the initial spreadsheet obtained to a VCF file. Because of that the real disease causing variant was not present in the VCF file which was initially inserted to Mendel,MD. After correcting the initial conversion script we were able to correctly identify the gene and variant for these clinical cases.

Case 8 we had one individual P5, after using 1-Click we received 26 genes, being two already present at OMIM, we select the gene *FARS2* [geneid:10667] with two heterozygous mutations in chromosome 6 at positions 5545494 and 5613508 both sift score of 0, polyphen2 score of 1.0, and CADD score of 4.29 and 4.18 respectively.

Case 9 was represented by one individual P3 affected by a compound heterozygous disease. We used 1-Click selecting this type of inheritance and we got 46 genes and 4 associated with a disease at OMIM. After considering the list of symptoms, the gene *TK2*[geneid:7084] was selected as the best candidate. This gene had two mutations, one at the position 16:66551110 - a non-synonymous mutation with a sift score of 0 and Polyphen2 score of 0.97. The other mutation at the position 16:66551095 had a sift score of 0.15, a polyphen2 score of 0.05, but a CADD raw score of 3.44, which is a good reason to select those variants as being the most likely to cause the disease in this clinical case.

In Case 10, was represented by a patient with a dominant disorder. After using 1-Click functionality of Mendel,MD we limited a list of 261 genes to 51 genes already present at OMIM. After comparing the list of symptoms with the disease described we identified a dominant mutation in gene *GJC2*[geneid:57165]. This mutation at position 1:228345602 had a sift score of 0.14, polyphen2 score of 0.95 and a CADD score of 4.01.

Case 11 was represented by a trio, where the child had a recessive disorder. After using 1-Click method we got only 8 genes, one already present at OMIM. Because the gene did not match the symptoms we reduced the read depth threshold to 5 and then we identified 31 genes, from which 6 already were present at OMIM. From the list of 6 genes we selected *KIF1A*[geneid:547] gene as being the best candidate. The gene had a mutation at 2:241723190 position which had a sift score of 0.00, polyphen2 score of 0.60 and a CADD score of 3.56. This case is an example of why we should always check the coverage of individuals before performing eliminating variants. The child in this case had mean of coverage of 23.52X which is a good indication that the minimum read depth needs to be reduced.

This clinical cases demonstrated the good performance of Mendel,MD and indicated that the tool can be used to help with the investigation of Mendelian Disorders with a certain attention for the right parameters which should be adjusted for each clinical case, based on a set of information such as the read depth and quality of the sequencing. BAM files should always be used for visualization of the region of interest.

On S1 Table we present statistics about all the received exomes used for validation. Here it’s possible to check the number of variants and the mean of coverage for each case.

This information is very useful to define thresholds while performing variant filtering and prioritization. After looking at these metrics we defined thresholds such as a minimum read depth of 5 for exomes with average coverage of 30 and a minimum read depth of 10 for exomes with average of coverage that were higher than 30.
