# Supplementary material for: Mendel,MD: A user-friendly open-source web tool for analyzing WES and WGS in the diagnosis of patients with Mendelian disorders
Source: PLoS Comput Biol. 2017 Jun 8;13(6):e1005520. doi: 10.1371/journal.pcbi.1005520 (PMC5464533; doi:10.1371/journal.pcbi.1005520)
Supplement: S1 Code — Last version of the source-code of Mendel,MD. (ZIP) [file pcbi.1005520.s004.zip › mendelmd-master/mendelmd_source/apps/cases/templates/cases/list.html]

{% extends "base.html" %}
{% load i18n %}
{% load paginator %}
{% block title %}{% trans "List Cases" %}{% endblock %}
{% block content %}

# Cases

{% trans "Create a new Case" %}

  
{% if cases %}

| id | status | name | description | members | Options |
| --- | --- | --- | --- | --- | --- |
{% for case in cases %}| {{case.id}} | {{case.status}} | {{case.name}} | {{case.description}} | Mother:{{case.mother.name}}  Father:{{case.father.name}}  Children:  {% for individual in case.children.all %} {{individual.name}}  {% endfor %}  Cases: {% for individual in case.cases.all %} {{individual.name}}  {% endfor %}  Controls: {% for individual in case.controls.all %} {{individual.name}}  {% endfor %} | View Edit Delete |
{% endfor %}

{% else %}
You still have no cases created.   
Please create your cases into the system!
{% endif %}
{% endblock %}
