# Supplementary material for: Mendel,MD: A user-friendly open-source web tool for analyzing WES and WGS in the diagnosis of patients with Mendelian disorders
Source: PLoS Comput Biol. 2017 Jun 8;13(6):e1005520. doi: 10.1371/journal.pcbi.1005520 (PMC5464533; doi:10.1371/journal.pcbi.1005520)
Supplement: S1 Code — Last version of the source-code of Mendel,MD. (ZIP) [file pcbi.1005520.s004.zip › mendelmd-master/mendelmd_source/apps/cases/templates/cases/new.html]

{% extends "base.html" %}
{% load i18n %}
{% load staticfiles %}
{% load django\_select2\_tags %}
{% block extra\_css %}


{% import\_django\_select2\_css %}
{% import\_django\_select2\_js %}
{% endblock %}
{% block title %}{% trans "Create Case" %}{% endblock %}
{% block content %}

# {% trans "Create Case" %}

{% csrf\_token %}

|  |  |
| --- | --- |
| status | {{form.status}} |
| name | {{form.name}} |
| description | {{form.description}} |
| Shared with | {{form.shared\_with}} |

|  |  |  |  |
| --- | --- | --- | --- |
| Father | {{form.father}} | Mother | {{form.mother}} |
| Children | {{form.children}} | | |

| Cases | Controls |
| --- | --- |
| {{form.cases}} | {{form.controls}} |
| {{form.case\_groups}} | {{form.control\_groups}} |

{% endblock %}
{% block javascript %}


{% endblock javascript %}
