# Supplementary material for: Mendel,MD: A user-friendly open-source web tool for analyzing WES and WGS in the diagnosis of patients with Mendelian disorders
Source: PLoS Comput Biol. 2017 Jun 8;13(6):e1005520. doi: 10.1371/journal.pcbi.1005520 (PMC5464533; doi:10.1371/journal.pcbi.1005520)
Supplement: S1 Code — Last version of the source-code of Mendel,MD. (ZIP) [file pcbi.1005520.s004.zip › mendelmd-master/mendelmd_source/apps/cases/templates/cases/view.html]

{% extends "base.html" %}
{% load i18n %}
{% load paginator %}
{% block title %}{% trans "View Case" %}{% endblock %}
{% block content %}

# Case {{case.id}} {{case.name}}

{% trans "Cases List" %}
{% trans "New Analysis" %}

  

| id | {{case.id}} |
| user | {{case.user}} |
| name | {{case.name}} |
| description | {{case.description}} |
| mother | {{case.mother}} |
| father | {{case.father}} |
| children | {% for individual in case.children.all %} {{individual.name}}   {% endfor %} |
| cases | {% for individual in case.cases.all %} {{individual.name}}  {% endfor %} |
| controls | {% for individual in case.controls.all %} {{individual.name}}  {% endfor %} |
| Options | Edit Delete |

## One Click

{% trans "One Click" %}

## Filter Analysis

{% trans "Autosomal Recessive" %}
{% trans "Recessive Compound Heterozygous" %}
{% trans "Dominant Heterozygous" %}
{% trans "X-Linked" %}

## Family Analysis

{% trans "Recessive Homozygous" %}
{% trans "Recessive Compound Heterozygous" %}
{% trans "Dominant Heterozygous" %}
{% trans "X-Linked" %}

## Pathway Analysis

{% trans "Recessive Homozygous" %}
{% trans "Recessive Compound Heterozygous" %}
{% trans "Dominant Heterozygous" %}
{% trans "X-Linked" %}

{% endblock %}
