# Supplementary material for: Mendel,MD: A user-friendly open-source web tool for analyzing WES and WGS in the diagnosis of patients with Mendelian disorders
Source: PLoS Comput Biol. 2017 Jun 8;13(6):e1005520. doi: 10.1371/journal.pcbi.1005520 (PMC5464533; doi:10.1371/journal.pcbi.1005520)
Supplement: S1 Code — Last version of the source-code of Mendel,MD. (ZIP) [file pcbi.1005520.s004.zip › mendelmd-master/mendelmd_source/apps/dashboard/templates/dashboard/dashboard.html]

{% extends "base.html" %}
{% block content %}

# Dashboard

Upload VCF File
  
  

{% csrf\_token %}
{% if user.is\_staff %}
Show on Grid
Hide on Grid
Delete
Populate
Annotate

{% endif %}

## Individuals

| # | ID | Name |{% if user.is\_staff %} Options |{% endif %} Uploaded By | Nº Lines | Created on | Modified on | Annotation Time | Insertion Time | Status |{% if user.is\_staff %} Operations |{% endif %}
| --- | --- | --- | --- | --- | --- | --- | --- | --- | --- | --- | --- |
{% for individual in individuals %}|  | {{ individual.id }} | {{ individual.name|truncatechars:30 }} |{% if user.is\_staff %} - Edit - Delete |{% endif %} {{ individual.user.username }} | {{ individual.n\_lines }} | {{ individual.creation\_date }} | {{ individual.modified\_date }} | {{ individual.annotation\_time }} | {{ individual.insertion\_time }} | {{ individual.status }} |{% if user.is\_staff %} *Reannotate*  *Repopulate* |{% endif %}
{% endfor %}

{% endblock %}
