# Supplementary material for: Mendel,MD: A user-friendly open-source web tool for analyzing WES and WGS in the diagnosis of patients with Mendelian disorders
Source: PLoS Comput Biol. 2017 Jun 8;13(6):e1005520. doi: 10.1371/journal.pcbi.1005520 (PMC5464533; doi:10.1371/journal.pcbi.1005520)
Supplement: S1 Code — Last version of the source-code of Mendel,MD. (ZIP) [file pcbi.1005520.s004.zip › mendelmd-master/mendelmd_source/apps/databases/templates/databases/index.html]

{% extends "base.html" %}
{% load i18n %}
{% block title %}{% trans "Databases Admin" %}{% endblock %}
{% block content %}

# {% trans "Database Admin" %}

### Genes

- Populate Genes from hgnc
- Populate Diseases from omim
- Populate Omim Genes coordinates from refseq
- Populate Clinical Genomics Database
- Populate VariSNP
- Create CGD Lupski Gene Lists

{% endblock %}
