# Supplementary material for: Mendel,MD: A user-friendly open-source web tool for analyzing WES and WGS in the diagnosis of patients with Mendelian disorders
Source: PLoS Comput Biol. 2017 Jun 8;13(6):e1005520. doi: 10.1371/journal.pcbi.1005520 (PMC5464533; doi:10.1371/journal.pcbi.1005520)
Supplement: S1 Code — Last version of the source-code of Mendel,MD. (ZIP) [file pcbi.1005520.s004.zip › mendelmd-master/mendelmd_source/apps/diseases/templates/diseases/disease_list.html]

{% extends "base.html" %}
{% load i18n %}
{% load pagination\_tags %}
{% block title %}{% trans "Diseases" %}{% endblock %}
{% block content %}

# {% trans "Diseases" %}

Search
{% if diseases %}
{% if is\_paginated %}

{% if page\_obj.has\_previous %}
previous
{% endif %}
Page {{ page\_obj.number }} of {{ page\_obj.paginator.num\_pages }}.
{% if page\_obj.has\_next %}
next
{% endif %}

{% endif %}
{{ page\_obj.paginator.count }} Diseases
{% csrf\_token %}

| # | Name | Genes |
| --- | --- | --- |
{% for disease in diseases %}|  | {{ disease.name }} | {{ disease.gene\_names }} |
{% endfor %}

{% if is\_paginated %}

{% if page\_obj.has\_previous %}
previous
{% endif %}
Page {{ page\_obj.number }} of {{ page\_obj.paginator.num\_pages }}.
{% if page\_obj.has\_next %}
next
{% endif %}

{% endif %}
{% else %}

No diseases found!!! :(

{% endif %}
{% endblock %}
{% block extra\_script %}
{% endblock %}
