# Supplementary material for: Mendel,MD: A user-friendly open-source web tool for analyzing WES and WGS in the diagnosis of patients with Mendelian disorders
Source: PLoS Comput Biol. 2017 Jun 8;13(6):e1005520. doi: 10.1371/journal.pcbi.1005520 (PMC5464533; doi:10.1371/journal.pcbi.1005520)
Supplement: S1 Code — Last version of the source-code of Mendel,MD. (ZIP) [file pcbi.1005520.s004.zip › mendelmd-master/mendelmd_source/apps/diseases/templates/diseases/list.html]

{% extends "site\_base.html" %}
{% load i18n %}
{% load pagination\_tags %}
{% block head\_title %}{% trans "Diseases" %}{% endblock %}
{% block body %}

# {% trans "Diseases" %}


{% for disease in diseases %}- {{ disease.name }} {{ disease.gene }}
{% endfor %}
{% endblock %}
