# Supplementary material for: Mendel,MD: A user-friendly open-source web tool for analyzing WES and WGS in the diagnosis of patients with Mendelian disorders
Source: PLoS Comput Biol. 2017 Jun 8;13(6):e1005520. doi: 10.1371/journal.pcbi.1005520 (PMC5464533; doi:10.1371/journal.pcbi.1005520)
Supplement: S1 Code — Last version of the source-code of Mendel,MD. (ZIP) [file pcbi.1005520.s004.zip › mendelmd-master/mendelmd_source/apps/diseases/templates/diseases/view.html]

{% extends "full\_base.html" %}
{% load i18n %}
{% block title %}{% trans "View Disease" %}{% endblock %}
{% block content %}

# {{ disease.name }}

## {% trans "Gene" %}

{% for gene in disease.gene\_set.all %}- Names: {{ gene.names }}
{% endfor %}

## {% trans "Individual Variants" %}

{% include "variants.html" %}
{% endblock %}
