# Supplementary material for: Mendel,MD: A user-friendly open-source web tool for analyzing WES and WGS in the diagnosis of patients with Mendelian disorders
Source: PLoS Comput Biol. 2017 Jun 8;13(6):e1005520. doi: 10.1371/journal.pcbi.1005520 (PMC5464533; doi:10.1371/journal.pcbi.1005520)
Supplement: S1 Code — Last version of the source-code of Mendel,MD. (ZIP) [file pcbi.1005520.s004.zip › mendelmd-master/mendelmd_source/apps/filter_analysis/templates/analysis/filter_form.html]

- Individuals
- Variants
- Databases
- Diseases
- Saved Configs
- Saved Analysis
- FAQ
- Fields

| Select Variants From | | Exclude Variants From | |
| --- | --- | --- | --- |
| {{ form.individuals.errors }}{{ form.individuals.label }}:   {{ form.individuals }}   {{ form.snp\_list.errors }} {{ form.snp\_list.label }}:  {{ form.snp\_list }} | {{ form.groups.label }}:   {{ form.groups }}   {{ form.genelists.errors }} {{ form.genelists.label }}:   {{ form.genelists }}   {{ form.gene\_list.errors }} {{ form.gene\_list.label }}:   {{ form.gene\_list }} | {{ form.exclude\_individuals.errors }} {{ form.exclude\_individuals.label }}:   {{ form.exclude\_individuals }}  {{ form.exclude\_snp\_list.label }}:  {{ form.exclude\_snp\_list }} | {{ form.exclude\_groups.label }}:   {{ form.exclude\_groups }}   {{ form.exclude\_genelists.errors }} {{ form.exclude\_genelists.label }}:   {{ form.exclude\_genelists }}   {{ form.exclude\_gene\_list.errors }} {{ form.exclude\_gene\_list.label }}:   {{ form.exclude\_gene\_list }} |

|  |  |  |
| --- | --- | --- |
| {{ form.mutation\_type.errors }} {{ form.mutation\_type.label }}: {{ form.mutation\_type }} | {{ form.chr.errors }} {{ form.chr.label }}: {{ form.chr }}   {{ form.pos.errors }} {{ form.pos.label }}: {{ form.pos }} | {{ form.filter }} |
| Variant Effect | Functional Class | Impact |
| {{ form.variant\_type }} | {{ form.func\_class }} | {{ form.impact }} |
| {{ form.dbsnp\_option.label }}:   {{ form.dbsnp\_option }} {{ form.dbsnp\_build }} | {{ form.read\_depth\_option.errors }} {{ form.read\_depth\_option.label }}:   {{ form.read\_depth\_option }} {{ form.read\_depth.errors }}{{ form.read\_depth }}   {{ form.qual\_option.errors }} {{ form.qual\_option.label }}:   {{ form.qual\_option }} {{ form.qual.errors }}{{ form.qual }} | {{ form.variants\_per\_gene\_option.errors }} {{ form.variants\_per\_gene\_option.label }}:   {{ form.variants\_per\_gene\_option }} {{ form.variants\_per\_gene.errors }}{{ form.variants\_per\_gene }} |
| {{ form.genes\_in\_common.errors }} {{ form.genes\_in\_common }} {{ form.genes\_in\_common.label }}   {{ form.positions\_in\_common.errors }} {{ form.positions\_in\_common }} {{ form.positions\_in\_common.label }}   {{ form.dbsnp.errors }} {{ form.dbsnp }} {{ form.dbsnp.label }}   {{ form.cln.errors }} {{ form.cln }} {{ form.cln.label }}   {{ form.exclude\_segdup.errors }} {{ form.exclude\_segdup }} {{ form.exclude\_segdup.label }} | | |

|  |  |
| --- | --- |
| OMIM | {{ form.omim }} |
| CGD | {{ form.cgd }} |
| CGD Manifestation | {{ form.cgdmanifestation }} |
| HGMD | {{ form.hgmd }} |

{% for filterconfig in filterconfigs %}| Name | User | Created on | Options |
| --- | --- | --- | --- |
| {{ filterconfig.name }} | {{ filterconfig.user }} | {{ filterconfig.created }} | EditDelete |
{% endfor %}

{% for filteranalysis in filteranalysis %}| Name | User | Created on | Options |
| --- | --- | --- | --- |
| {{ filteranalysis.name }} | {{ filteranalysis.user }} | {{ filteranalysis.created }} | EditDelete |
{% endfor %}

|  |  |
| --- | --- |
| 1000Genomes Frequency | {{ form.genomes1000.errors }}{{ form.genomes1000 }}      {{form.genomes1000\_exclude}}{{form.genomes1000\_exclude.label}} |
| dbSNP Frequency | {{ form.dbsnp\_frequency.errors }}{{ form.dbsnp\_frequency }}        {{form.dbsnp\_exclude}}{{form.dbsnp\_exclude.label}} |
| Exome Variation Server Frequency | {{ form.esp\_frequency.errors }}{{ form.esp\_frequency }}        {{form.esp\_exclude}}{{form.esp\_exclude.label}} |
| Sift Score  \*from snpEff | {{ form.sift.errors }} {{ form.sift }}        {{ form.sift\_exclude.errors }} {{ form.sift\_exclude }}{{ form.sift\_exclude.label }} |
| Polyphen Score | {{ form.polyphen.errors }}{{ form.polyphen }}        {{ form.polyphen\_exclude }}{{ form.polyphen\_exclude.label }} |
| Haploinsufficiency | {{ form.hi\_frequency.errors }}{{ form.hi\_frequency }}        {{ form.hi\_exclude }}{{ form.hi\_exclude.label }} |

Example of genotype information:

0/1:0.45:10,12:22:99:211,0,262
  

**GT:AB:AD:DP:GQ:PL**

**GT: Genotype**

Genotype, encoded as allele values separated by either of ”/” or “|”. The allele values are 0 for the reference allele (what is in the REF field), 1 for the first allele listed in ALT, 2 for the second allele list in ALT and so on. For diploid calls examples could be 0/1, 1|0, or 1/2, etc. For haploid calls, e.g. on Y, male non-pseudoautosomal X, or mitochondrion, only one allele value should be given; a triploid call might look like 0/0/1. If a call cannot be made for a sample at a given locus, ”.” should be specified for each missing allele in the GT field (for example "./." for a diploid genotype and "." for haploid genotype). The meanings of the separators are as follows (see the PS field below for more details on incorporating phasing information into the genotypes)

**AB: Allele balance for each het genotype**

The allele balance (fraction of ref bases over ref + alt bases) across all bialleleic het-called samples

**AD: Allelic depths for the ref and alt alleles in the order listed**

The depth of coverage of each VCF allele in this sample.

**DP: Read Depth (only filtered reads used for calling)**
**GQ: Genotype Quality**

Conditional genotype quality, encoded as a phred quality -10log\_10p(genotype call is wrong, conditioned on the site's being variant) (Float)

**PL: Normalized, Phred-scaled likelihoods for genotypes as defined in the VCF specification**

The phred-scaled genotype likelihoods rounded to the closest integer (and otherwise defined precisely as the GL field) (Integers)

{{ form.fields }}

Open result in a new window

Reset Filter
{% if query\_string %}
| Save Config
| Save Analysis
{% endif %}
