# Supplementary material for: Mendel,MD: A user-friendly open-source web tool for analyzing WES and WGS in the diagnosis of patients with Mendelian disorders
Source: PLoS Comput Biol. 2017 Jun 8;13(6):e1005520. doi: 10.1371/journal.pcbi.1005520 (PMC5464533; doi:10.1371/journal.pcbi.1005520)
Supplement: S1 Code — Last version of the source-code of Mendel,MD. (ZIP) [file pcbi.1005520.s004.zip › mendelmd-master/mendelmd_source/apps/filter_analysis/templates/filter_analysis/family_analysis.html]

{% extends "base.html" %}
{% load staticfiles %}
{% load humanize %}
{% load i18n %}
{#% load sorting\_tags %#}
{% load pagination\_tags %}
{% load filter\_extras %}
{% block title %}{% trans "Family Analysis" %}{% endblock %}
{% block extra\_css %}


{% endblock %}
{% block content %}

# Family Analysis

#### + Filter Options

{% include "filter\_analysis/filter\_form\_familyanalysis.html" %}

#### + Genes {% if summary.genes %}{{summary.genes|length}}{% endif %}

Genes:
  
{% for gene in summary.genes %}
{{ gene }},
{% endfor %}

#### + Genes associated with diseases {% if genes\_omim %}{{genes\_omim|length}}{% endif %}

{% include "tabs/genes.html" %}

{%if variants %}

#### Summary

Number of Variants: {{ summary.n\_variants }}
  
Number of Genes: {{ summary.n\_genes }}

{% endif %}
{% if variants %}
Export to: CSV
  
{% include "pagination.html" %}

{% include "tabs/variants\_familyanalysis.html" %}

{% include "pagination.html" %}
{% endif %}
{% endblock %}
{% block javascript %}

{% for variant in variants.object\_list %}
{% endfor %}
{% endblock javascript %}
