# Supplementary material for: Mendel,MD: A user-friendly open-source web tool for analyzing WES and WGS in the diagnosis of patients with Mendelian disorders
Source: PLoS Comput Biol. 2017 Jun 8;13(6):e1005520. doi: 10.1371/journal.pcbi.1005520 (PMC5464533; doi:10.1371/journal.pcbi.1005520)
Supplement: S1 Code — Last version of the source-code of Mendel,MD. (ZIP) [file pcbi.1005520.s004.zip › mendelmd-master/mendelmd_source/apps/filter_analysis/templates/filter_analysis/filter_form.html]

- Main
- Variants
- Databases
- Diseases
- Saved Configs
- Saved Analysis
- FAQ

{% include "tabs/main.html" %}

{% include "tabs/variants.html" %}

{% include "tabs/databases.html" %}

{% include "tabs/diseases.html" %}

{% include "tabs/filter\_config.html" %}

{% include "tabs/filter\_analysis.html" %}

{% include "tabs/faq.html" %}

OPEN RESULT IN A NEW WINDOW

RESET FILTER
{% if query\_string %}
| Save Config
| Save Analysis
{% endif %}
