# Supplementary material for: Mendel,MD: A user-friendly open-source web tool for analyzing WES and WGS in the diagnosis of patients with Mendelian disorders
Source: PLoS Comput Biol. 2017 Jun 8;13(6):e1005520. doi: 10.1371/journal.pcbi.1005520 (PMC5464533; doi:10.1371/journal.pcbi.1005520)
Supplement: S1 Code — Last version of the source-code of Mendel,MD. (ZIP) [file pcbi.1005520.s004.zip › mendelmd-master/mendelmd_source/apps/filter_analysis/templates/filter_analysis/table.html]

{% extends "base.html" %}
{% load staticfiles %}
{% load django\_select2\_tags %}
{% load i18n %}
{% load sorting\_tags %}
{% load pagination\_tags %}
{% load filter\_extras %}
{% block title %}{% trans "Filter Analysis - Table" %}{% endblock %}
{% block extra\_css %}


{% import\_django\_select2\_css %}
{% import\_django\_select2\_js %}
{% endblock %}
{% block content %}

|  |  |  |  |
| --- | --- | --- | --- |
| Options | Variants | Genes | Genes at OMIM |
{% for row in table %}|{% for key, value in row.items %} {{key}} |{% for key2, item in value.items %} {{item}} |{% endfor %}
{% endfor %}
{% endfor %}

{% endblock %}
{% block javascript %}

{% for variant in variants.object\_list %}
{% endfor %}
{% endblock javascript %}
