# Supplementary material for: Mendel,MD: A user-friendly open-source web tool for analyzing WES and WGS in the diagnosis of patients with Mendelian disorders
Source: PLoS Comput Biol. 2017 Jun 8;13(6):e1005520. doi: 10.1371/journal.pcbi.1005520 (PMC5464533; doi:10.1371/journal.pcbi.1005520)
Supplement: S1 Code — Last version of the source-code of Mendel,MD. (ZIP) [file pcbi.1005520.s004.zip › mendelmd-master/mendelmd_source/apps/filter_analysis/templates/filteranalysis/Copy of index.html]

{% extends "site\_base.html" %}
{% load bootstrap\_tags %}
{% load humanize %}
{% load staticfiles %}
{% block extra\_head %}
{% endblock %}
{% load i18n %}
{% load sorting\_tags %}
{% load pagination\_tags %}
{% load filter\_extras %}
{% block head\_title %}{% trans "Filter Analysis" %}{% endblock %}
{% block body %}

Filtering

- Individuals
- Basic
- Genes
- Databases
- Scores
- Impact
- Diseases
- Saved Configs
- Saved Analysis
- Other Options
- FAQ

|  |  |  |  |
| --- | --- | --- | --- |
| {{ form.individuals.errors }} {{ form.individuals.label }}:   {{ form.individuals }}   {{ form.inheritance\_model.errors }} {{ form.inheritance\_model.label }}:   {{ form.inheritance\_model }}   {{ form.groups.label }}:   {{ form.groups }} | {{ form.exclude\_individuals.errors }} {{ form.exclude\_individuals.label }}:   {{ form.exclude\_individuals }}   {{ form.exclude\_groups.label }}:   {{ form.exclude\_groups }} |  |  |

|  |  |
| --- | --- |
| Pre-defined Configs:     Standard 1 Other Option Other Option Other Option   {{ form.non\_field\_errors }} {{ form.chr.errors }} {{ form.chr.label }}:{{ form.chr }}   {{ form.pos.errors }} {{ form.pos.label }}:{{ form.pos }}   {{ form.snp\_list.errors }} {{ form.snp\_list.label }}:   {{ form.snp\_list }} | {{ form.exclude\_snp\_list.label }}:   {{ form.exclude\_snp\_list }} |

{% for filterconfig in filterconfigs %}| Name | User | Created on | Options |
| --- | --- | --- | --- |
| {{ filterconfig.name }} | {{ filterconfig.user }} | {{ filterconfig.created }} | EditDelete |
{% endfor %}

{% for filteranalysis in filteranalysis %}| Name | User | Created on | Options |
| --- | --- | --- | --- |
| {{ filteranalysis.name }} | {{ filteranalysis.user }} | {{ filteranalysis.created }} | EditDelete |
{% endfor %}

|  |  |  |
| --- | --- | --- |
| Create Gene Group   {{ form.genegroups.errors }} {{ form.genegroups.label }}:   {{ form.genegroups }}   {{ form.gene\_list.errors }} {{ form.gene\_list.label }}:   {{ form.gene\_list }} | {{ form.exclude\_gene\_list.errors }} {{ form.exclude\_gene\_list.label }}:   {{ form.exclude\_gene\_list }} |  |

{{ form.genomes1000\_option.errors }}
{{ form.genomes1000\_option.label }}:
  
{{ form.genomes1000\_option }}
{{ form.genomes1000.errors }}{{ form.genomes1000 }}
  
{{ form.dbsnp\_freq\_option.errors }}
{{ form.dbsnp\_freq\_option.label }}:
  
{{ form.dbsnp\_freq\_option }}
{{ form.dbsnp\_frequency.errors }}{{ form.dbsnp\_frequency }}
  
{{ form.variationserver\_option.errors }}
{{ form.variationserver\_option.label }}:
  
{{ form.variationserver\_option }}
{{ form.variationserver\_frequency.errors }}{{ form.variationserver\_frequency }}

{{ form.sift\_option.errors }}
{{ form.sift\_option.label }}:
  
{{ form.sift\_option }}
{{ form.sift.errors }}{{ form.sift }}
  
{{ form.sift\_exclude.errors }}
{{ form.sift\_exclude }}{{ form.sift\_exclude.label }}
  
{{ form.polyphen\_option.errors }}
{{ form.polyphen\_option.label }}:
  
{{ form.polyphen\_option }}
{{ form.polyphen.errors }}{{ form.polyphen }}
  
{{ form.polyphen\_exclude.errors }}
{{ form.polyphen\_exclude }}{{ form.polyphen\_exclude.label }}

{{ form.variant\_type.errors }}
{{ form.variant\_type.label }}:
  
{{ form.variant\_type }}
{{ form.func\_class.errors }}
  
{{ form.func\_class.label }}:
  
{{ form.func\_class }}
{{ form.impact.errors }}
  
{{ form.impact.label }}:
  
{{ form.impact }}

{{ form.diseases.errors }}
  
{{ form.diseases.label }}:
  
{{ form.diseases }}

{{ form.read\_depth\_option.errors }}
{{ form.read\_depth\_option.label }}:
  
{{ form.read\_depth\_option }}
{{ form.read\_depth.errors }}{{ form.read\_depth }}
  
{{ form.variants\_per\_gene\_option.errors }}
{{ form.variants\_per\_gene\_option.label }}:
  
{{ form.variants\_per\_gene\_option }}
{{ form.variants\_per\_gene.errors }}{{ form.variants\_per\_gene }}
  
{{ form.genes\_in\_common.errors }}
{{ form.genes\_in\_common }}
  
{{ form.genes\_in\_common.label }}
  
{{ form.dbsnp.errors }}
{{ form.dbsnp }}
  
{{ form.dbsnp.label }}
  
{{ form.cln.errors }}
{{ form.cln }}
  
{{ form.cln.label }}

Example of genotype information:

0/1:0.45:10,12:22:99:211,0,262
  

**GT:AB:AD:DP:GQ:PL**

**GT: Genotype**

Genotype, encoded as allele values separated by either of ”/” or “|”. The allele values are 0 for the reference allele (what is in the REF field), 1 for the first allele listed in ALT, 2 for the second allele list in ALT and so on. For diploid calls examples could be 0/1, 1|0, or 1/2, etc. For haploid calls, e.g. on Y, male non-pseudoautosomal X, or mitochondrion, only one allele value should be given; a triploid call might look like 0/0/1. If a call cannot be made for a sample at a given locus, ”.” should be specified for each missing allele in the GT field (for example "./." for a diploid genotype and "." for haploid genotype). The meanings of the separators are as follows (see the PS field below for more details on incorporating phasing information into the genotypes)

**AB: Allele balance for each het genotype**

The allele balance (fraction of ref bases over ref + alt bases) across all bialleleic het-called samples

**AD: Allelic depths for the ref and alt alleles in the order listed**

The depth of coverage of each VCF allele in this sample.

**DP: Read Depth (only filtered reads used for calling)**
**GQ: Genotype Quality**

Conditional genotype quality, encoded as a phred quality -10log\_10p(genotype call is wrong, conditioned on the site's being variant) (Float)

**PL: Normalized, Phred-scaled likelihoods for genotypes as defined in the VCF specification**

The phred-scaled genotype likelihoods rounded to the closest integer (and otherwise defined precisely as the GL field) (Integers)

Open result in a new window
  

{% if query\_string %} Save Analysis
  
Save Config {% endif %}

Summary

Genes:
  
{% for gene in summary.genes %} {{ gene.gene\_name }} (Gene Variants),
{% endfor %}

Reset Filter
{%if summary.n\_variants %}

#### Summary

Number of Variants: {{ summary.n\_variants }}
  
Number of Genes: {{ summary.n\_genes }}

{% endif %}

{% if variants %}
Export to: CSVTXT
{% autosort variants %}

{% if variants.has\_previous %}- ‹‹ {% trans "previous" %}
{% else %}- ‹‹ {% trans "previous" %}
{% endif %}
{% for page in variants.paginator.page\_range %}
{% if page %}
{% ifequal page variants.number %}- {{ page }}
{% else %} 
{% if page|adjust\_for\_pagination:variants.number %}- {{ page }}
{% endif %}
{% endifequal %}
{% else %}
{% endif %}
{% endfor %}
{% if variants.has\_next %}- {% trans "next" %} ››
{% else %}- {% trans "next" %} ››
{% endif %}

| {% anchor individual Individual %} | {% anchor chromossome Chr %} | {% anchor id RsId %} | {% anchor pos Pos %} | {% anchor qual Qual %} | {% anchor ref Ref %} | {% anchor alt Alt %} | {% anchor genotype Gen %} | {% anchor read\_depth "Read Depth" %} | {% anchor snp\_eff "Mutation Type"" %} | {% anchor snp\_eff\_functional\_class "Protein Impact" %} | {% anchor impact "SnpEff Class" %} | {% anchor genomes1k\_af "1kgenomes" %} | {% anchor dbsnp\_gmaf "dbSNP137" %} | {% anchor ann\_esp\_maf "ESP6500" %} | {% anchor sift Sift %} | {% anchor polyphen Polyphen %} | Other Info |
| --- | --- | --- | --- | --- | --- | --- | --- | --- | --- | --- | --- | --- | --- | --- | --- | --- | --- |
{% regroup variants.object\_list by gene\_name as gene\_list %}
{% for gene in gene\_list %}
{% if gene.grouper %}| {{ gene.grouper }} |
{% endif %}| Omim - GeneCards - NCBI | | |
{% for variant in gene.list %}| {{ variant.individual }} | {{ variant.chromossome }} | {{ variant.variant\_id }} {% if variant.variant\_id != "." %}   dbSNP |{% endif %} {{ variant.pos }} | {{ variant.qual }} | {{ variant.ref }} | {{ variant.alt }} | {{ variant.genotype }} | {{ variant.read\_depth }} | {{ variant.snp\_eff|cleanstr }} | {{ variant.snp\_eff\_functional\_class }} | {{ variant.impact }} | {% if variant.genomes1k\_af != None %} {{ variant.genomes1k\_af }} {% endif %} | {{ variant.gmaf\_dbsnp135|floatformat:5|intcomma }} | {{ variant.ann\_esp\_maf|floatformat:5|intcomma }} | {{ variant.sift|floatformat:2 }} | {{ variant.polyphen|floatformat:2 }} | View More |
{% endfor %}
{% endfor %}

{% endif %}
{% endblock %}
{% block extra\_js %}
{% for variant in variants.object\_list %}
{% endfor %}
{% endblock %} 
