# Supplementary material for: Mendel,MD: A user-friendly open-source web tool for analyzing WES and WGS in the diagnosis of patients with Mendelian disorders
Source: PLoS Comput Biol. 2017 Jun 8;13(6):e1005520. doi: 10.1371/journal.pcbi.1005520 (PMC5464533; doi:10.1371/journal.pcbi.1005520)
Supplement: S1 Code — Last version of the source-code of Mendel,MD. (ZIP) [file pcbi.1005520.s004.zip › mendelmd-master/mendelmd_source/apps/filter_analysis/templates/filteranalysis/index.html]

{% extends "base.html" %}
{% load staticfiles %}
{% load i18n %}
{% load pagination\_tags %}
{% load filter\_extras %}
{% block extra\_css %}
{% endblock %}
{% block title %}{% trans "Filter Analysis" %}{% endblock %}
{% block content %}

#### + Filter Options

{% include "filter\_analysis/filter\_form\_oneclick.html" %}

#### + Genes {% if summary.genes %}{{summary.genes|length}}{% endif %}

Genes:
  
{% for gene in summary.genes %}
{{ gene }},
{% endfor %}

#### + Genes associated with diseases {% if genes\_omim %}{{genes\_omim|length}}{% endif %}

{% for gene in genes %}|  |  |
| --- | --- |
|{% if gene.diseases.all %} {{ gene.symbol }} | {% for disease in gene.diseases.all %} {{disease.name}}  {% endfor %} |{% endif %}
{% endfor %}

### Genes at Omim

{% for gene in genes\_omim %}|  |  |
| --- | --- |
| {{ gene.official\_name}} | {% for disease in gene.diseases.all %} {{disease.name}}  {% endfor %} |
{% endfor %}

### Genes at Clinical Genomics Database

{% for gene in genes\_cgd %}|  |  |
| --- | --- |
| {{gene.GENE}} | {% for phenotype in gene.CONDITIONS.all %} {{phenotype.name}}  {% endfor %} |
{% endfor %}

### Genes at HGMD

{% for gene in genes\_hgmd %}|  |  |
| --- | --- |
| {{gene.symbol}} | {% for phenotype in gene.diseases.all %} {{phenotype.name}}  {% endfor %} |
{% endfor %}

{%if summary.n\_variants %}

#### Summary

Number of Variants: {{ summary.n\_variants }}
  
Number of Genes: {{ summary.n\_genes }}

{% endif %}
{% if summary.has\_variants %}
Export to: CSV
  
{% include "pagination.html" %}

{% include "variants.html" %}

{% include "pagination.html" %}
{% endif %}
{% endblock %}
{% block extra\_js %}
{% endblock %}
