# Supplementary material for: Mendel,MD: A user-friendly open-source web tool for analyzing WES and WGS in the diagnosis of patients with Mendelian disorders
Source: PLoS Comput Biol. 2017 Jun 8;13(6):e1005520. doi: 10.1371/journal.pcbi.1005520 (PMC5464533; doi:10.1371/journal.pcbi.1005520)
Supplement: S1 Code — Last version of the source-code of Mendel,MD. (ZIP) [file pcbi.1005520.s004.zip › mendelmd-master/mendelmd_source/apps/filter_analysis/templates/forms/oneclick.html]

{% extends "base.html" %}
{% block content %}

Individuals

{% csrf\_token %}

| Select Variants From | | Exclude Variants From | |
| --- | --- | --- | --- |
| {{ form.individuals.errors }}{{ form.individuals.label }}:   {{ form.individuals }}   {{ form.snp\_list.errors }} {{ form.snp\_list.label }}:  {{ form.snp\_list }} | {{ form.groups.label }}:   {{ form.groups }}   {{ form.gene\_list.errors }} {{ form.gene\_list.label }}:   {{ form.gene\_list }} | {{ form.exclude\_individuals.errors }} {{ form.exclude\_individuals.label }}:   {{ form.exclude\_individuals }}  {{ form.exclude\_snp\_list.label }}:  {{ form.exclude\_snp\_list }} | {{ form.exclude\_groups.label }}:   {{ form.exclude\_groups }}   {{ form.exclude\_gene\_list.errors }} {{ form.exclude\_gene\_list.label }}:   {{ form.exclude\_gene\_list }} |

Collapsible Group Item #2

Anim pariatur cliche reprehenderit, enim eiusmod high life accusamus terry richardson ad squid. 3 wolf moon officia aute, non cupidatat skateboard dolor brunch. Food truck quinoa nesciunt laborum eiusmod. Brunch 3 wolf moon tempor, sunt aliqua put a bird on it squid single-origin coffee nulla assumenda shoreditch et. Nihil anim keffiyeh helvetica, craft beer labore wes anderson cred nesciunt sapiente ea proident. Ad vegan excepteur butcher vice lomo. Leggings occaecat craft beer farm-to-table, raw denim aesthetic synth nesciunt you probably haven't heard of them accusamus labore sustainable VHS.

Collapsible Group Item #3

Anim pariatur cliche reprehenderit, enim eiusmod high life accusamus terry richardson ad squid. 3 wolf moon officia aute, non cupidatat skateboard dolor brunch. Food truck quinoa nesciunt laborum eiusmod. Brunch 3 wolf moon tempor, sunt aliqua put a bird on it squid single-origin coffee nulla assumenda shoreditch et. Nihil anim keffiyeh helvetica, craft beer labore wes anderson cred nesciunt sapiente ea proident. Ad vegan excepteur butcher vice lomo. Leggings occaecat craft beer farm-to-table, raw denim aesthetic synth nesciunt you probably haven't heard of them accusamus labore sustainable VHS.

{{ previous\_fields|safe }}


{% endblock %}
