# Supplementary material for: Mendel,MD: A user-friendly open-source web tool for analyzing WES and WGS in the diagnosis of patients with Mendelian disorders
Source: PLoS Comput Biol. 2017 Jun 8;13(6):e1005520. doi: 10.1371/journal.pcbi.1005520 (PMC5464533; doi:10.1371/journal.pcbi.1005520)
Supplement: S1 Code — Last version of the source-code of Mendel,MD. (ZIP) [file pcbi.1005520.s004.zip › mendelmd-master/mendelmd_source/apps/filter_analysis/templates/forms/step2.html]

{% extends "base.html" %}
{% load staticfiles %}
{% block extra\_head %}


{% endblock %}
{% block content %}
{% csrf\_token %}

Step {{ step }} of {{ step\_count }}

|  |  |
| --- | --- |
| 1000Genomes Frequency | {{ form.genomes1000.errors }}{{ form.genomes1000 }}        {{form.genomes1000\_exclude}}{{form.genomes1000\_exclude.label}} |
| dbSNP Frequency | {{ form.dbsnp\_frequency.errors }}{{ form.dbsnp\_frequency }}         {{form.dbsnp\_exclude}}{{form.dbsnp\_exclude.label}} |
| Exome Variation Server Frequency | {{ form.esp\_frequency.errors }}{{ form.esp\_frequency }}         {{form.esp\_exclude}}{{form.esp\_exclude.label}} |
| Sift Score | {{ form.sift\_option }} {{ form.sift.errors }}{{ form.sift }}   {{ form.sift\_exclude.errors }} {{ form.sift\_exclude }}{{ form.sift\_exclude.label }} |
| Polyphen Score | {{ form.polyphen\_option }} {{ form.polyphen.errors }}{{ form.polyphen }}         {{ form.polyphen\_exclude.errors }} {{ form.polyphen\_exclude }}{{ form.polyphen\_exclude.label }} |
| Mendel,MD Score | {{ form.mendelmd\_score }} {{ form.mendelmd\_score.errors }} |

{{ previous\_fields|safe }}
{% endblock %}
{% block extra\_js %}
{% endblock %} 
