# Supplementary material for: Mendel,MD: A user-friendly open-source web tool for analyzing WES and WGS in the diagnosis of patients with Mendelian disorders
Source: PLoS Comput Biol. 2017 Jun 8;13(6):e1005520. doi: 10.1371/journal.pcbi.1005520 (PMC5464533; doi:10.1371/journal.pcbi.1005520)
Supplement: S1 Code — Last version of the source-code of Mendel,MD. (ZIP) [file pcbi.1005520.s004.zip › mendelmd-master/mendelmd_source/apps/filter_analysis/templates/tabs/databases.html]

### FREQUENCIES

|  |  |  |  |  |  |
| --- | --- | --- | --- | --- | --- |
| 1000 GENOMES FREQUENCY | {{ form.genomes1000.errors }}{{ form.genomes1000 }}      {{form.genomes1000\_exclude}}{{form.genomes1000\_exclude.label}} | DBSNP FREQUENCY | {{ form.dbsnp\_frequency.errors }}{{ form.dbsnp\_frequency }}        {{form.dbsnp\_exclude}}{{form.dbsnp\_exclude.label}} | ESP6500 FREQUENCY | {{ form.esp\_frequency.errors }}{{ form.esp\_frequency }}        {{form.esp\_exclude}}{{form.esp\_exclude.label}} |

### SCORES

|  |  |  |  |
| --- | --- | --- | --- |
| SIFT SCORE | {{ form.sift.errors }} {{ form.sift }}        {{ form.sift\_exclude.errors }} {{ form.sift\_exclude }}{{ form.sift\_exclude.label }} | POLYPHEN2 SCORE | {{ form.polyphen.errors }}{{ form.polyphen }}        {{ form.polyphen\_exclude }}{{ form.polyphen\_exclude.label }} |
| CADD | {{ form.cadd.errors }}{{ form.cadd }}      {{ form.cadd\_exclude }}{{ form.cadd\_exclude.label }} |
