# Supplementary material for: Mendel,MD: A user-friendly open-source web tool for analyzing WES and WGS in the diagnosis of patients with Mendelian disorders
Source: PLoS Comput Biol. 2017 Jun 8;13(6):e1005520. doi: 10.1371/journal.pcbi.1005520 (PMC5464533; doi:10.1371/journal.pcbi.1005520)
Supplement: S1 Code — Last version of the source-code of Mendel,MD. (ZIP) [file pcbi.1005520.s004.zip › mendelmd-master/mendelmd_source/apps/filter_analysis/templates/tabs/faq.html]

Example of genotype information:

0/1:0.45:10,12:22:99:211,0,262
  

**GT:AB:AD:DP:GQ:PL**

**GT: Genotype**

Genotype, encoded as allele values separated by either of ”/” or “|”. The allele values are 0 for the reference allele (what is in the REF field), 1 for the first allele listed in ALT, 2 for the second allele list in ALT and so on. For diploid calls examples could be 0/1, 1|0, or 1/2, etc. For haploid calls, e.g. on Y, male non-pseudoautosomal X, or mitochondrion, only one allele value should be given; a triploid call might look like 0/0/1. If a call cannot be made for a sample at a given locus, ”.” should be specified for each missing allele in the GT field (for example "./." for a diploid genotype and "." for haploid genotype). The meanings of the separators are as follows (see the PS field below for more details on incorporating phasing information into the genotypes)

**AB: Allele balance for each het genotype**

The allele balance (fraction of ref bases over ref + alt bases) across all bialleleic het-called samples

**AD: Allelic depths for the ref and alt alleles in the order listed**

The depth of coverage of each VCF allele in this sample.

**DP: Read Depth (only filtered reads used for calling)**
**GQ: Genotype Quality**

Conditional genotype quality, encoded as a phred quality -10log\_10p(genotype call is wrong, conditioned on the site's being variant) (Float)

**PL: Normalized, Phred-scaled likelihoods for genotypes as defined in the VCF specification**

The phred-scaled genotype likelihoods rounded to the closest integer (and otherwise defined precisely as the GL field) (Integers)
