# Supplementary material for: Mendel,MD: A user-friendly open-source web tool for analyzing WES and WGS in the diagnosis of patients with Mendelian disorders
Source: PLoS Comput Biol. 2017 Jun 8;13(6):e1005520. doi: 10.1371/journal.pcbi.1005520 (PMC5464533; doi:10.1371/journal.pcbi.1005520)
Supplement: S1 Code — Last version of the source-code of Mendel,MD. (ZIP) [file pcbi.1005520.s004.zip › mendelmd-master/mendelmd_source/apps/filter_analysis/templates/tabs/filter_analysis.html]

{% for filteranalysis in filteranalysis %}| Name | User | Created on | Options |
| --- | --- | --- | --- |
| {{ filteranalysis.name }} | {{ filteranalysis.user }} | {{ filteranalysis.created }} | EditDelete |
{% endfor %}
