# Supplementary material for: Mendel,MD: A user-friendly open-source web tool for analyzing WES and WGS in the diagnosis of patients with Mendelian disorders
Source: PLoS Comput Biol. 2017 Jun 8;13(6):e1005520. doi: 10.1371/journal.pcbi.1005520 (PMC5464533; doi:10.1371/journal.pcbi.1005520)
Supplement: S1 Code — Last version of the source-code of Mendel,MD. (ZIP) [file pcbi.1005520.s004.zip › mendelmd-master/mendelmd_source/apps/filter_analysis/templates/tabs/genes.html]

### Genes at Omim

{% for gene in genes\_omim %}
{{ gene.official\_name }},
{% endfor %}

{% for gene in genes\_omim %}|  |  |
| --- | --- |
| {{ gene.official\_name }} | {% for disease in gene.diseases.all %} {{disease.name}}  {% endfor %} |
{% endfor %}

### Genes at Clinical Genomics Database

{% for gene in genes\_cgd %}
{{ gene.GENE }},
{% endfor %}

{% for gene in genes\_cgd %}|  |  |
| --- | --- |
| {{ gene.GENE }} | {% for phenotype in gene.CONDITIONS.all %} {{phenotype.name}}  {% endfor %} |
{% endfor %}

{% for gene in genes\_hgmd %}
{{ gene.symbol }}
{% endfor %}

{% for gene in genes\_hgmd %}|  |  |
| --- | --- |
| {{ gene.symbol }} | {% for phenotype in gene.diseases.all %} {{phenotype.name}}  {% endfor %} |
{% endfor %}
