# Supplementary material for: Mendel,MD: A user-friendly open-source web tool for analyzing WES and WGS in the diagnosis of patients with Mendelian disorders
Source: PLoS Comput Biol. 2017 Jun 8;13(6):e1005520. doi: 10.1371/journal.pcbi.1005520 (PMC5464533; doi:10.1371/journal.pcbi.1005520)
Supplement: S1 Code — Last version of the source-code of Mendel,MD. (ZIP) [file pcbi.1005520.s004.zip › mendelmd-master/mendelmd_source/apps/filter_analysis/templates/tabs/main_oneclick.html]

| SELECT VARIANTS FROM | | EXCLUDE VARIANTS FROM | |
| --- | --- | --- | --- |
| {{ form.individuals.errors }}{{ form.individuals.label }}:   {{ form.individuals }}   {{ form.snp\_list.errors }} {{ form.snp\_list.label }}:  {{ form.snp\_list }} | {{ form.groups.label }}:   {{ form.groups }}   {{ form.genelists.errors }} {{ form.genelists.label }}:   {{ form.genelists }}   {{ form.gene\_list.errors }} {{ form.gene\_list.label }}:   {{ form.gene\_list }} | {{ form.exclude\_individuals.errors }} {{ form.exclude\_individuals.label }}:   {{ form.exclude\_individuals }}  {{ form.exclude\_snp\_list.label }}:  {{ form.exclude\_snp\_list }} | {{ form.exclude\_groups.label }}:   {{ form.exclude\_groups }}   {{ form.exclude\_genelists.errors }} {{ form.exclude\_genelists.label }}:   {{ form.exclude\_genelists }}   {{ form.exclude\_gene\_list.errors }} {{ form.exclude\_gene\_list.label }}:   {{ form.exclude\_gene\_list }} |
| SELECT INHERITANCE: RECESSIVE HOMOZYGOUS RECESSIVE COMPOUND HETEROZYGOUS DOMINANT HETEROZYGOUS X-LINKED RECESSIVE HEMIZYGOUS X-LINKED DOMINANT HETEROZYGOUS | | | |
| **SELECT YOUR DISEASES:** | | | |
| OMIM: {{form.omim}} | | | |
| CLINICAL GENOMICS DATABASE: {{form.cgd}} | | | |
| HGMD: {{form.hgmd}} | | | |
