# Supplementary material for: Mendel,MD: A user-friendly open-source web tool for analyzing WES and WGS in the diagnosis of patients with Mendelian disorders
Source: PLoS Comput Biol. 2017 Jun 8;13(6):e1005520. doi: 10.1371/journal.pcbi.1005520 (PMC5464533; doi:10.1371/journal.pcbi.1005520)
Supplement: S1 Code — Last version of the source-code of Mendel,MD. (ZIP) [file pcbi.1005520.s004.zip › mendelmd-master/mendelmd_source/apps/filter_analysis/templates/tabs/variants_pathwayanalysis.html]

|  |  |  |
| --- | --- | --- |
| {{ form.mutation\_type.errors }} {{ form.mutation\_type.label }}: {{ form.mutation\_type }} | {{ form.chr.errors }} {{ form.chr.label }}: {{ form.chr }}   {{ form.pos.errors }} {{ form.pos.label }}: {{ form.pos }} | {{ form.filter }} |
| Variant Effect | Functional Class | Impact |
| {{ form.variant\_type }} | {{ form.func\_class }} | {{ form.impact }} |
| {{ form.dbsnp\_option.label }}:   {{ form.dbsnp\_option }} {{ form.dbsnp\_build }} | {{ form.read\_depth\_option.errors }} {{ form.read\_depth\_option.label }}:   {{ form.read\_depth\_option }} {{ form.read\_depth.errors }}{{ form.read\_depth }}   {{ form.qual\_option.errors }} {{ form.qual\_option.label }}:   {{ form.qual\_option }} {{ form.qual.errors }}{{ form.qual }} | {{ form.variants\_per\_gene\_option.errors }} {{ form.variants\_per\_gene\_option.label }}:   {{ form.variants\_per\_gene\_option }} {{ form.variants\_per\_gene.errors }}{{ form.variants\_per\_gene }}   {{ form.individuals\_per\_pathway\_option.label }}:  {{ form.individuals\_per\_pathway\_option }} {{ form.individuals\_per\_pathway }} |
| {{ form.genes\_in\_common.errors }} {{ form.genes\_in\_common }} {{ form.genes\_in\_common.label }}   {{ form.positions\_in\_common.errors }} {{ form.positions\_in\_common }} {{ form.positions\_in\_common.label }}   {{ form.dbsnp.errors }} {{ form.dbsnp }} {{ form.dbsnp.label }}   {{ form.cln.errors }} {{ form.cln }} {{ form.cln.label }}   {{ form.exclude\_segdup.errors }} {{ form.exclude\_segdup }} {{ form.exclude\_segdup.label }} | | |
