# Supplementary material for: Mendel,MD: A user-friendly open-source web tool for analyzing WES and WGS in the diagnosis of patients with Mendelian disorders
Source: PLoS Comput Biol. 2017 Jun 8;13(6):e1005520. doi: 10.1371/journal.pcbi.1005520 (PMC5464533; doi:10.1371/journal.pcbi.1005520)
Supplement: S1 Code — Last version of the source-code of Mendel,MD. (ZIP) [file pcbi.1005520.s004.zip › mendelmd-master/mendelmd_source/apps/genes/templates/genes/gene_list.html]

{% extends "base.html" %}
{% load i18n %}
{% block title %}{% trans "List Genes" %}{% endblock %}
{% block content %}

# Gene Lists

Create GeneList

| Name | Options |
| --- | --- |
{% for genelist in genelists %}| {{genelist.name}} | View Delete |
{% endfor %}

# {% trans "Genes" %}

{% include "search.html" %}

{% if page\_obj.has\_previous %}
previous
{% endif %}
Page {{ page\_obj.number }} of {{ page\_obj.paginator.num\_pages }}.
{% if page\_obj.has\_next %}
next
{% endif %}

{% csrf\_token %}

| # | Symbol | Name | Options |
| --- | --- | --- | --- |
{% for gene in genes %}|  | {{ gene.symbol }} | {{ gene.name }} | View Gene Variants |
{% endfor %}

{% include "pagination.html" %}
{% endblock %}
{% block extra\_js %}
{% endblock %}
