# Supplementary material for: Mendel,MD: A user-friendly open-source web tool for analyzing WES and WGS in the diagnosis of patients with Mendelian disorders
Source: PLoS Comput Biol. 2017 Jun 8;13(6):e1005520. doi: 10.1371/journal.pcbi.1005520 (PMC5464533; doi:10.1371/journal.pcbi.1005520)
Supplement: S1 Code — Last version of the source-code of Mendel,MD. (ZIP) [file pcbi.1005520.s004.zip › mendelmd-master/mendelmd_source/apps/genes/templates/genes/geneontology_view.html]

{% extends "site\_base.html" %}
{% load i18n %}
{% load gene\_extras %}
{% block head\_title %}{% trans "Gene Ontology" %}{% endblock %}
{% block body %}

# {% trans "Gene Ontology" %}

{{ goterm.goid }} - {{ goterm.name }}

## Parents

{% for goterm in parents.all %}- {{ goterm.name }}
{% endfor %}

## Children

{% for goterm in children.all %}- {{ goterm.name }}
{% endfor %}
{% if genes %}

## Genes

{% if genes.has\_previous %}- previous
{% else %}- ‹‹ {% trans "previous" %}
{% endif %}
{% for page in genes.paginator.page\_range %}
{% if page %}
{% ifequal page genes.number %}- {{ page }}
{% else %} 
{% if page|adjust\_for\_pagination:genes.number %}- {{ page }}
{% endif %}
{% endifequal %}
{% else %}
{% endif %}
{% endfor %}
{% if genes.has\_next %}- {% trans "next" %} ››
{% else %}- {% trans "next" %} ››
{% endif %}

{% for gene in genes %}- {{gene.gene.symbol}}
{% endfor %}
{% endif %}
{% endblock %}
