# Supplementary material for: Mendel,MD: A user-friendly open-source web tool for analyzing WES and WGS in the diagnosis of patients with Mendelian disorders
Source: PLoS Comput Biol. 2017 Jun 8;13(6):e1005520. doi: 10.1371/journal.pcbi.1005520 (PMC5464533; doi:10.1371/journal.pcbi.1005520)
Supplement: S1 Code — Last version of the source-code of Mendel,MD. (ZIP) [file pcbi.1005520.s004.zip › mendelmd-master/mendelmd_source/apps/genes/templates/genes/view.html]

{% extends "base.html" %}
{% load gene\_extras %}
{% load i18n %}
{% block title %}{% trans "Gene" %}{% endblock %}
{% block content %}

# {% trans "Gene" %} - {{ gene\_object.symbol }}

- Gene Description
- Variants

{% for field, value in gene\_object.get\_fields %}|  |  |
| --- | --- |
| {{ field }} | {{ value }} |
{% endfor %}

## Variants By Individual

| Name | Novel | Known | Total Variants |
| --- | --- | --- | --- |
{% for individual in variants\_by\_individuals %}| {{ individual.individual }} | {{ individual.novel }} | {{ individual.known }} | {{ individual.total }} |
{% endfor %}| Total | {{variants\_summary.individual.novel}} | {{variants\_summary.individual.known}} | {{variants\_summary.individual.total}} |

## Variants by Effect

| Name |{% for item in dna\_variation\_classes %} {{ item }} |{% endfor %}
| --- | --- |
{% for individual,variants in individuals\_dna\_variation.items %}| {{ individual }} |{% for item in dna\_variation\_classes %} {% dictKeyLookup variants item %} |{% endfor %}
{% endfor %}| Total |{% for item in dna\_variation %} {{ item.total }} |{% endfor %}

## Variants By Functional Class

| Name |{% for item in functional\_class\_classes %} {{ item }} |{% endfor %}
| --- | --- |
{% for individual,variants in individuals\_functional\_class.items %}| {{ individual }} |{% for item in functional\_class\_classes %} {% dictKeyLookup variants item %} |{% endfor %}
{% endfor %}| Total |{% for item in functional\_class %} {{ item.total }} |{% endfor %}

## Variants By Impact

| Name |{% for item in impact\_classes %} {{ item }} |{% endfor %}
| --- | --- |
{% for individual,variants in individuals\_impact.items %}| {{ individual }} |{% for item in impact\_classes %} {% dictKeyLookup variants item %} |{% endfor %}
{% endfor %}| Total |{% for item in impact %} {{ item.total }} |{% endfor %}

Omim - GeneCards - NCBI
{% endblock %}
