# Supplementary material for: Mendel,MD: A user-friendly open-source web tool for analyzing WES and WGS in the diagnosis of patients with Mendelian disorders
Source: PLoS Comput Biol. 2017 Jun 8;13(6):e1005520. doi: 10.1371/journal.pcbi.1005520 (PMC5464533; doi:10.1371/journal.pcbi.1005520)
Supplement: S1 Code — Last version of the source-code of Mendel,MD. (ZIP) [file pcbi.1005520.s004.zip › mendelmd-master/mendelmd_source/apps/individuals/templates/individuals/comparison.html]

{% extends "base.html" %}
{% load i18n %}
{% load get\_genotype %}
{% block extra\_css %}
{% if summary %}
{% endif %}
{% endblock %}
{% block head\_title %}{% trans "View Group" %}{% endblock %}
{% block content %}

# {% trans "Comparison" %}

{{ form.as\_table }}

Number of variants in individual one:{{ summary.variants\_ind\_one }} - ({{ summary.percent\_ind\_one }}% in common)  
Number of variants in individual two:{{ summary.variants\_ind\_two }} - ({{ summary.percent\_ind\_two }}% in common)  
Number of variants in common: {{ summary.total\_variants }}{% endblock %}
