# Supplementary material for: Mendel,MD: A user-friendly open-source web tool for analyzing WES and WGS in the diagnosis of patients with Mendelian disorders
Source: PLoS Comput Biol. 2017 Jun 8;13(6):e1005520. doi: 10.1371/journal.pcbi.1005520 (PMC5464533; doi:10.1371/journal.pcbi.1005520)
Supplement: S1 Code — Last version of the source-code of Mendel,MD. (ZIP) [file pcbi.1005520.s004.zip › mendelmd-master/mendelmd_source/apps/individuals/templates/individuals/create.html]

{% extends "base.html" %}
{% load i18n %}
{% load upload\_tags %}
{% block extra\_css %}


{% endblock %}
{% block content %}

# Add VCFs

## Here you can upload your VCF files to the system. We support the following formats: VCF, VCF.GZ, VCF.ZIP and VCF.RAR

**The software presents features that ensure privacy of the data submitted. First, submitters are
provided with a username and password and only they can retrieve the clinical data. Second, names are not
required -- patients need only to be identified by a code name or number provided by the submitter. Users
who have not accessed the program in a period of three months, will be consulted by email to ascertain
whether they wish to maintain the data stored or whether it can be deleted. If there is no response in one
week, the data will be erased.
All data entered Mendel,MD in a public mode (without username and password) will be deleted in 24 hours.**

An exome VCF file should take around 20 minutes to be annotated and inserted to the database. A genome VCF file should take around 8 hours.  
You will receive an e-mail as soon this process finishes with the link to start analysing your individuals.

You can take this time to read our Documentation about how to filter your variants.

{% csrf\_token %}


Please, Select your VCF files...


Click here when your files have finished uploading!
{% upload\_js %}


{% endblock %}
{% block javascript %}
{% endblock %}
