# Supplementary material for: Mendel,MD: A user-friendly open-source web tool for analyzing WES and WGS in the diagnosis of patients with Mendelian disorders
Source: PLoS Comput Biol. 2017 Jun 8;13(6):e1005520. doi: 10.1371/journal.pcbi.1005520 (PMC5464533; doi:10.1371/journal.pcbi.1005520)
Supplement: S1 Code — Last version of the source-code of Mendel,MD. (ZIP) [file pcbi.1005520.s004.zip › mendelmd-master/mendelmd_source/apps/individuals/templates/individuals/list.html]

{% extends "base.html" %}
{% load paginator %}
{% block content %}

# Individuals

Upload VCF Files
Create a new Group

  
{% if individuals %}

{% for individual in ind\_featured %}
{% if individual.is\_featured %}

{{ individual.name }}

{% endif %}
{% endfor %}

{% endif %}
{% if groups %}

# Groups

| # | Group Name | Members | Options |
| --- | --- | --- | --- |
{% for group in groups %}|  | {{ group }} | {{ group.members.count }} | {% if user.is\_staff %} View, Edit,- Delete {% endif %} |
{% endfor %}

{% endif %}
{% if individuals %}

{% if show\_first %}- « First
{% endif %}
{% if has\_previous %}- ‹ Previous
{% endif %}
{% for one\_page in page\_numbers %}
{% ifequal one\_page page %}- {{ page }}
{% else %}- {{ one\_page }}
{% endifequal %}
{% endfor %}
{% if has\_next %}- Next ›
{% endif %}
{% if show\_last %}- Last »
{% endif %}
{% if individuals.has\_previous %}
previous
{% endif %}
Page {{ individuals.number }} of {{ individuals.paginator.num\_pages }}.
{% if individuals.has\_next %}
next
{% endif %}

{% csrf\_token %}
{{ form.as\_p }}
Show on Grid
Hide on Grid
Delete
Populate
Annotate
Find Medical Conditions and Medicines


| # | ID | Name | Options | Uploaded By | Nº Lines | Created on | Modified on | Annotation Time | Insertion Time | Insertion Time MongoDB | Status |{% if user.is\_staff %} Operations |{% endif %}
| --- | --- | --- | --- | --- | --- | --- | --- | --- | --- | --- | --- | --- |
{% for individual in individuals %}|  | {{ individual.id }} | {{ individual.name }} | - Edit - Browse - Delete | {{ individual.user.username }} | {{ individual.n\_lines }} | {{ individual.creation\_date }} | {{ individual.modified\_date }} | {{ individual.annotation\_time }} | {{ individual.insertion\_time }} | {{ individual.insertion\_time\_mongo }} | {{ individual.status }} |{% if user.is\_staff %} *Reannotate Individual*  *Repopulate Individual*  *Populate to MongoDB* |{% endif %}
{% endfor %}

{% else %}
You still have no individuals uploaded.   
Please upload your individuals to the system!
{% endif %}
{% endblock %}
{% block extra\_js %}
{% endblock %}
