# Supplementary material for: Mendel,MD: A user-friendly open-source web tool for analyzing WES and WGS in the diagnosis of patients with Mendelian disorders
Source: PLoS Comput Biol. 2017 Jun 8;13(6):e1005520. doi: 10.1371/journal.pcbi.1005520 (PMC5464533; doi:10.1371/journal.pcbi.1005520)
Supplement: S1 Code — Last version of the source-code of Mendel,MD. (ZIP) [file pcbi.1005520.s004.zip › mendelmd-master/mendelmd_source/apps/individuals/templates/individuals/view.html]

{% extends "base.html" %}
{% load i18n %}
{% load staticfiles %}
{% load pagination\_tags %}
{% load get\_genotype %}
{% block title %}{% trans "View Individual" %}{% endblock %}
{% block content %}

- Home
- Summary
- SNP Effect
- Functional Class
- Impact
- Filter
- Quality
- Read Depth
- Clinical Associated
- Variants per Chromossome

Name: {{ individual.name }}  
Variants: {{ individual.n\_variants }}  
Novel Variants: {{ individual.novel\_variants }} (Not in latest DbSNP Build)

- Download Original VCF File
- Download Annotated VCF File

| Type | Total Variants |  |
| --- | --- | --- |
{% for item in individual.summary %}| {{ item.type }} | {{ item.total }} |{% for value in item.discrete %} {{ value.total }} {{ value.genotype }} |{% endfor %}
{% endfor %}

| Type | Total Variants |
| --- | --- |
{% for item in individual.snp\_eff %}| {{ item.snpeff\_effect }} | {{ item.snpeff\_effect\_\_count }} |
{% endfor %}

| Type | Total Variants |
| --- | --- |
{% for item in individual.functional\_class %}| {{ item.snpeff\_func\_class }} | {{ item.snpeff\_func\_class\_\_count }} |
{% endfor %}

| Type | Total Variants |
| --- | --- |
{% for item in individual.impact\_variants %}| {{ item.snpeff\_impact }} | {{ item.snpeff\_impact\_\_count }} |
{% endfor %}

| Type | Total Variants |
| --- | --- |
{% for item in individual.filter\_variants %}| {{ item.filter }} | {{ item.filter\_\_count }} |
{% endfor %}

| Quality | Value |
| --- | --- |
| Min | {{ individual.quality.qual\_\_min }} |
| Average | {{ individual.quality.qual\_\_avg }} |
| Max | {{ individual.quality.qual\_\_max }} |

| Read Depth | Value |
| --- | --- |
| Min | {{ individual.read\_depth.read\_depth\_\_min }} |
| Average | {{ individual.read\_depth.read\_depth\_\_avg }} |
| Max | {{ individual.read\_depth.read\_depth\_\_max }} |

| Type | Total Variants |
| --- | --- |
{% for item in individual.clinvar\_clnsig %}| {{ item.clinvar\_clnsig }} | {{ item.total }} |
{% endfor %}

| Chromossome | Total Variants |
| --- | --- |
{% for item in individual.chromossome %}| {{ item.chr }} | {{ item.total }} |
{% endfor %}

{% for individual\_medical\_condition in individual\_medical\_conditions %}

## {{ individual\_medical\_condition.medical\_condition.name }}

  
{% autoescape off %}
{{ individual\_medical\_condition.medical\_condition.description\_html }}
{% endautoescape %}
{% for variant in individual\_medical\_condition.variants.all %}

## {{ variant.snp.name }}

{% autoescape off %}
{{ variant.snp.description\_html }}
{% endautoescape %}

## Individual Genotype: {{ variant.individual\_variant|get\_genotype }}, genoinfo:{{ variant.individual\_variant.genotype\_info }} qual: {{ variant.individual\_variant.qual }}

| Genotype | Magnitude | Summary |
| --- | --- | --- |
{% for snpgenotype in variant.snp.snpgenotype\_set.all %}| {{ snpgenotype.genotype }} | {{ snpgenotype.magnitude }} | {{ snpgenotype.summary }} |
{% endfor %}

{% endfor %}
{% endfor %}

{% for individual\_medicine in individual\_medicines %}

## {{ individual\_medicine.medicine.name }}

  
{% autoescape off %}
{{ individual\_medicine.medicine.description\_html }}
{% endautoescape %}
{% for variant in individual\_medicine.variants.all %}

## {{ variant.snp.name }}

{% autoescape off %}
{{ variant.snp.description\_html }}
{% endautoescape %}

## Individual Genotype: {{ variant.individual\_variant|get\_genotype }}, genoinfo:{{ variant.individual\_variant.genotype\_info }} qual: {{ variant.individual\_variant.qual }}

| Genotype | Magnitude | Summary |
| --- | --- | --- |
{% for snpgenotype in variant.snp.snpgenotype\_set.all %}| {{ snpgenotype.genotype }} | {{ snpgenotype.magnitude }} | {{ snpgenotype.summary }} |
{% endfor %}

{% endfor %}
{% endfor %}

{% endblock %}
