# Supplementary material for: Mendel,MD: A user-friendly open-source web tool for analyzing WES and WGS in the diagnosis of patients with Mendelian disorders
Source: PLoS Comput Biol. 2017 Jun 8;13(6):e1005520. doi: 10.1371/journal.pcbi.1005520 (PMC5464533; doi:10.1371/journal.pcbi.1005520)
Supplement: S1 Code — Last version of the source-code of Mendel,MD. (ZIP) [file pcbi.1005520.s004.zip › mendelmd-master/mendelmd_source/apps/pagination/templates/pagination/pagination.html]

Hello pagapp
{% if is\_paginated %}
{% load i18n %}

{% if page\_obj.has\_previous %}
‹‹ {% trans "previous" %}
{% else %}
‹‹ {% trans "previous" %}
{% endif %}
{% for page in pages %}
{% if page %}
{% ifequal page page\_obj.number %}
{{ page }}
{% else %}
{{ page }}
{% endifequal %}
{% else %}
...
{% endif %}
{% endfor %}
{% if page\_obj.has\_next %}
{% trans "next" %} ››
{% else %}
{% trans "next" %} ››
{% endif %}

{% endif %}
