# Supplementary material for: Mendel,MD: A user-friendly open-source web tool for analyzing WES and WGS in the diagnosis of patients with Mendelian disorders
Source: PLoS Comput Biol. 2017 Jun 8;13(6):e1005520. doi: 10.1371/journal.pcbi.1005520 (PMC5464533; doi:10.1371/journal.pcbi.1005520)
Supplement: S1 Code — Last version of the source-code of Mendel,MD. (ZIP) [file pcbi.1005520.s004.zip › mendelmd-master/mendelmd_source/apps/pathway_analysis/templates/pathway_analysis/analysis.html]

{% extends "base.html" %}
{% load staticfiles %}
{% load django\_select2\_tags %}
{% load i18n %}
{#% load sorting\_tags %#}
{% load pagination\_tags %}
{% load filter\_extras %}
{% block title %}{% trans "Filter Analysis" %}{% endblock %}
{% block extra\_css %}


{% import\_django\_select2\_css %}
{% import\_django\_select2\_js %}
{% endblock %}
{% block content %}

#### + Filter Options

{% include "filter\_analysis/filter\_form\_pathanalysis.html" %}

#### + Genes {% if summary.genes %}{{summary.genes|length}}{% endif %}

Genes:
  
{% if summary.n\_genes < 500 %}
{% for gene in summary.genes %}
{{ gene }},
{% endfor %}
{% else %}- Your list of genes is bigger than 500. Please, try to increase the parameters.
{% endif %}

#### + Genes associated with diseases {% if genes\_omim %}{{genes\_omim|length}}{% endif %}

{% include "tabs/genes.html" %}

{%if summary.n\_variants %}

#### Summary

Number of Variants: {{ summary.n\_variants }}
  
Number of Genes: {{ summary.n\_genes }}

{% endif %}
{% for pathway in pathways %}

#### {{pathway.name}} Variants: {{pathway.variants|length}}

View in Kegg
{% with pathway.variants as variants %}
{% include "variants.html" %}
{% endwith %}

{% endfor %}
{% endblock %}
{% block javascript %}

{% for pathway in pathways %}
{% for variant in pathway.variants %}
{% endfor %}
{% endfor %}
{% endblock javascript %}
