# Supplementary material for: Mendel,MD: A user-friendly open-source web tool for analyzing WES and WGS in the diagnosis of patients with Mendelian disorders
Source: PLoS Comput Biol. 2017 Jun 8;13(6):e1005520. doi: 10.1371/journal.pcbi.1005520 (PMC5464533; doi:10.1371/journal.pcbi.1005520)
Supplement: S1 Code — Last version of the source-code of Mendel,MD. (ZIP) [file pcbi.1005520.s004.zip › mendelmd-master/mendelmd_source/apps/pathway_analysis/templates/pathway_analysis/filterform_header.html]

{% load staticfiles %}
{% load django\_select2\_tags %}
{% block extra\_css %}


{% import\_django\_select2\_css %}
{% import\_django\_select2\_js %}
{% endblock %}
