# Supplementary material for: Mendel,MD: A user-friendly open-source web tool for analyzing WES and WGS in the diagnosis of patients with Mendelian disorders
Source: PLoS Comput Biol. 2017 Jun 8;13(6):e1005520. doi: 10.1371/journal.pcbi.1005520 (PMC5464533; doi:10.1371/journal.pcbi.1005520)
Supplement: S1 Code — Last version of the source-code of Mendel,MD. (ZIP) [file pcbi.1005520.s004.zip › mendelmd-master/mendelmd_source/apps/pathway_analysis/templates/pathway_analysis/pathway_filterform.html]

#### + Filter Options

{% include "filter\_analysis/filter\_form\_pathanalysis.html" %}

#### + Genes {% if summary.genes %}{{summary.genes|length}}{% endif %}

Genes:
  
{% if summary.n\_genes < 500 %}
{% for gene in summary.genes %}
{{ gene }},
{% endfor %}
{% else %}- Your list of genes is bigger than 500. Please, try to increase the parameters.
{% endif %}

#### + Genes associated with diseases {% if genes\_omim %}{{genes\_omim|length}}{% endif %}

{% include "tabs/genes.html" %}
