# Supplementary material for: Mendel,MD: A user-friendly open-source web tool for analyzing WES and WGS in the diagnosis of patients with Mendelian disorders
Source: PLoS Comput Biol. 2017 Jun 8;13(6):e1005520. doi: 10.1371/journal.pcbi.1005520 (PMC5464533; doi:10.1371/journal.pcbi.1005520)
Supplement: S1 Code — Last version of the source-code of Mendel,MD. (ZIP) [file pcbi.1005520.s004.zip › mendelmd-master/mendelmd_source/apps/variants/templates/full_variants.html]

  

{% if variants.has\_previous %}
previous
{% endif %}
Page {{ variants.number }} of {{ variants.paginator.num\_pages }}.
{% if variants.has\_next %}
next
{% endif %}

|  |  |  |  |  |  |  |  |  |  |  |  |  |  |  |  |  |
| --- | --- | --- | --- | --- | --- | --- | --- | --- | --- | --- | --- | --- | --- | --- | --- | --- |
| options | index | chr | pos | variant\_id | ref | alt | qual | filter | info | format | genotype\_col | genotype | read\_depth | gene | mutation\_type | vartype |
{% for variant in variants.object\_list %}| View | {{ variant.index }} | {{ variant.chr }} | {{ variant.pos }} | {{ variant.variant\_id }} | {{ variant.ref\|truncatechars:5 }} | {{ variant.alt\|truncatechars:5 }} | {{ variant.qual }} | {{ variant.filter }} | - | {{ variant.format }} | {{ variant.genotype\_col }} | {{ variant.genotype }} | {{ variant.read\_depth }} | {{ variant.gene }} | {{ variant.mutation\_type }} | {{ variant.vartype }} |
|  | {% if variant.effect %} effect \| impact \| functional\_class \| codon\_change \| aa\_change \| aa\_len \| gene\_name \| biotype \| gene\_coding \| transcript\_id \| exon\_rank \| genotype\_number; {{ variant.effect }} \| {{ variant.impact }} \| {{ variant.func\_class }} \| {{ variant.codon\_change }} \| {{ variant.aa\_change }} \| {{ variant.aa\_len }} \| {{ variant.gene\_name }} \| {{ variant.biotype }} \| {{ variant.gene\_coding }} \| {{ variant.transcript\_id }} \| {{ variant.exon\_rank }} \| {{ variant.genotype\_number }} {% endif %} genomes1k\_maf \| dbsnp\_maf \| esp\_maf \| dbsnp\_build; {{ variant.genomes1k\_maf }} \| {{ variant.dbsnp\_maf }} \| {{ variant.esp\_maf }} \| {{ variant.dbsnp\_build }} sift \| sift\_pred \| polyphen2 \| polyphen2\_pred \| condel \| condel\_pred \| dann \| cadd \| is\_at\_omim \| is\_at\_hgmd \| hgmd\_entries; {{ variant.sift }} \| {{ variant.sift\_pred }} \| {{ variant.polyphen2 }} \| {{ variant.polyphen2\_pred }} \| {{ variant.condel }} \| {{ variant.condel\_pred }} \| {{ variant.dann }} \| {{ variant.cadd }} \| {{ variant.is\_at\_omim }} \| {{ variant.is\_at\_hgmd }} \| {{ variant.hgmd\_entries }} | | | | | | | | | | | | | | | |

{% endfor %}

{% if variants.has\_previous %}
previous
{% endif %}
Page {{ variants.number }} of {{ variants.paginator.num\_pages }}.
{% if variants.has\_next %}
next
{% endif %}
