# Supplementary material for: Mendel,MD: A user-friendly open-source web tool for analyzing WES and WGS in the diagnosis of patients with Mendelian disorders
Source: PLoS Comput Biol. 2017 Jun 8;13(6):e1005520. doi: 10.1371/journal.pcbi.1005520 (PMC5464533; doi:10.1371/journal.pcbi.1005520)
Supplement: S1 Code — Last version of the source-code of Mendel,MD. (ZIP) [file pcbi.1005520.s004.zip › mendelmd-master/mendelmd_source/apps/variants/templates/variants/view.html]

{% extends "base.html" %}
{% block content %}

# View Variant {{ variant.id }}

- Variant
- INFO
- SNPEFF
- VEP

|  |  |
| --- | --- |
| individual | {{ variant.individual.id }} - {{ variant.individual }} |
| index | {{ variant.index }} |
| chr | {{ variant.chr }} |
| pos | {{ variant.pos }} |
| variant\_id | {{ variant.variant\_id }} |
| ref | {{ variant.ref }} |
| alt | {{ variant.alt }} |
| qual | {{ variant.qual }} |
| filter | {{ variant.filter }} |
| format | {{ variant.format }} |
| genotype\_col | {{ variant.genotype\_col }} |
| genotype | {{ variant.genotype }} |
| read\_depth | {{ variant.read\_depth }} |
| gene | {{ variant.gene }} |
| mutation\_type | {{ variant.mutation\_type }} |
| vartype | {{ variant.vartype }} |
| genomes1k\_maf | {{ variant.genomes1k\_maf }} |
| dbsnp\_maf | {{ variant.dbsnp\_maf }} |
| esp\_maf | {{ variant.esp\_maf }} |
| dbsnp\_build | {{ variant.dbsnp\_build }} |
| sift | {{ variant.sift }} |
| sift\_pred | {{ variant.sift\_pred }} |
| polyphen2 | {{ variant.polyphen2 }} |
| polyphen2\_pred | {{ variant.polyphen2\_pred }} |
| condel | {{ variant.condel }} |
| condel\_pred | {{ variant.condel\_pred }} |
| dann | {{ variant.dann }} |
| cadd | {{ variant.cadd }} |
| is\_at\_omim | {{ variant.is\_at\_omim }} |
| is\_at\_hgmd | {{ variant.is\_at\_hgmd }} |
| hgmd\_entries | {{ variant.hgmd\_entries }} |

# INFO

{% for key, value in variant.info.items %}|  |  |
| --- | --- |
| {{key}} | {{value}} |
{% endfor %}

# snpEff

|  |  |
| --- | --- |
| effect | {{ variant.snpeff\_effect }} |
| impact | {{ variant.snpeff\_impact }} |
| functional\_class | {{ variant.snpeff\_func\_class }} |
| codon\_change | {{ variant.snpeff\_codon\_change }} |
| aa\_change | {{ variant.snpeff\_aa\_change }} |
| aa\_len | {{ variant.snpeff\_aa\_len }} |
| gene\_name | {{ variant.snpeff\_gene\_name }} |
| biotype | {{ variant.snpeff\_biotype }} |
| gene\_coding | {{ variant.snpeff\_gene\_coding }} |
| transcript\_id | {{ variant.snpeff\_transcript\_id }} |
| exon\_rank | {{ variant.snpeff\_exon\_rank }} |
| genotype\_number | {{ variant.snpeff\_genotype\_number }} |
|  |  |

# VEP

|  |  |
| --- | --- |
| allele | {{variant.vep\_allele}} |
| gene | {{variant.vep\_gene}} |
| feature | {{variant.vep\_feature}} |
| feature\_type | {{variant.vep\_feature\_type}} |
| consequence | {{variant.vep\_consequence}} |
| cdna\_position | {{variant.vep\_cdna\_position}} |
| cds\_position | {{variant.vep\_cds\_position}} |
| protein\_position | {{variant.vep\_protein\_position}} |
| amino\_acids | {{variant.vep\_amino\_acids}} |
| codons | {{variant.vep\_codons}} |
| existing\_variation | {{variant.vep\_existing\_variation}} |
| distance | {{variant.vep\_distance}} |
| strand | {{variant.vep\_strand}} |
| symbol | {{variant.vep\_symbol}} |
| symbol\_source | {{variant.vep\_symbol\_source}} |
| sift | {{variant.vep\_sift}} |
| polyphen | {{variant.vep\_polyphen}} |
| condel | {{variant.vep\_condel}} |

{% endblock content %}
