# Supplementary material for: Mendel,MD: A user-friendly open-source web tool for analyzing WES and WGS in the diagnosis of patients with Mendelian disorders
Source: PLoS Comput Biol. 2017 Jun 8;13(6):e1005520. doi: 10.1371/journal.pcbi.1005520 (PMC5464533; doi:10.1371/journal.pcbi.1005520)
Supplement: S1 Code — Last version of the source-code of Mendel,MD. (ZIP) [file pcbi.1005520.s004.zip › mendelmd-master/mendelmd_source/static/docs/admin_guide.html]

<no title> — Mendel,MD 0.3 documentation


### Related Topics

- Documentation overview

### This Page

- Show Source

### Quick search

©2017, Raony Guimaraes.
|
Powered by Sphinx 1.5.5
& Alabaster 0.7.10
|
Page source
