# Supplementary material for: Mendel,MD: A user-friendly open-source web tool for analyzing WES and WGS in the diagnosis of patients with Mendelian disorders
Source: PLoS Comput Biol. 2017 Jun 8;13(6):e1005520. doi: 10.1371/journal.pcbi.1005520 (PMC5464533; doi:10.1371/journal.pcbi.1005520)
Supplement: S1 Code — Last version of the source-code of Mendel,MD. (ZIP) [file pcbi.1005520.s004.zip › mendelmd-master/mendelmd_source/static/docs/annotation.html]

Pynnotator — Mendel,MD 0.3 documentation


# Pynnotator¶

A Python Annotation Framework for VCF files (Exome or Genome) from
Humans using multiple tools and databases.

# Libraries¶

- htslib (1.3.1)
- vcftools (0.1.14)
- snpeff (SnpEff 4.3)
- vep (version 84)

# Databases¶

- 1000Genomes (Phase 3) -
  ALL.wgs.phase3\_shapeit2\_mvncall\_integrated\_v5b.20130502.sites.vcf
- dbSNP (including clinvar) - (human\_9606\_b147\_GRCh37p13)
- Exome Sequencing Project - ESP6500SI-V2-SSA137.GRCh38-liftover
- dbNFSP 3.2a (including dbscSNV 1.1) -
- Ensembl 84 (phenotype and clinically associated variants)

# Features¶

- Multithread Efficient
- Annotate a VCF file using multiple VCFs as a reference

# Requirements¶

- Ubuntu 16.04 (probably also works on 14.04)
- Python 3.4+
- At least 40GB

# Installation¶

```
mkvirtualenv -p /usr/bin/python3 pynnotator
pip install cython
pip install pynnotator
pynnotator install
```

# Test¶

```
pynnotator test
```

### Table Of Contents

- Pynnotator
- Libraries
- Databases
- Features
- Requirements
- Installation
- Test

### Related Topics

- Documentation overview
  - Previous: Mendel,MD
  - Next: Clinical Cases

### This Page

- Show Source

### Quick search

©2017, Raony Guimaraes.
|
Powered by Sphinx 1.5.5
& Alabaster 0.7.10
|
Page source
