# Supplementary material for: Mendel,MD: A user-friendly open-source web tool for analyzing WES and WGS in the diagnosis of patients with Mendelian disorders
Source: PLoS Comput Biol. 2017 Jun 8;13(6):e1005520. doi: 10.1371/journal.pcbi.1005520 (PMC5464533; doi:10.1371/journal.pcbi.1005520)
Supplement: S1 Code — Last version of the source-code of Mendel,MD. (ZIP) [file pcbi.1005520.s004.zip › mendelmd-master/mendelmd_source/static/docs/clinical_cases.html]

Clinical Cases — Mendel,MD 0.3 documentation


# Clinical Cases¶

We prepared some VCF files to demonstrate how to perform variant priorization using the 1-Click method of Mendel,MD

http://mendel.medicina.ufmg.br/filter\_analysis/oneclick/

The four VCF files to be uploaded are inside the ‘examples’ folder of Mendel,MD.

We used the exome of individual NA12878 from the 1000Genomes Project and included variants found in previous cases studied by our own laboratory.

Before doing this validation you need to upload the VCF files and wait for them to be annotated.

## Upload of Examples¶

1. Go to http://mendel.medicina.ufmg.br/individuals/create/ and upload the four VCF files.
2. Wait all four VCFs to be annotated and inserted at the database.
3. Visit the “Dashboard” section to know the status of each VCF. Once their status show as ‘populated’ you can proceed with the analysis.
4. Click the “Submit” button.

## Autosomal Recessive - Homozygous¶

1. Go to http://mendel.medicina.ufmg.br/filter\_analysis/oneclick/
2. Add individual ‘NA12878 recessive’ to section SELECT VARIANTS FROM - INDIVIDUALS
3. Select Inheritance “Recessive Homozygous”
4. Click the “Submit” button.

Summary:
Number of Variants: 17
Number of Genes: 12
Number of Genes at OMIM: 1

The only gene found at OMIM is gene “SUCLA2” already known to be associated with “Mitochondrial DNA depletion syndrome 5 (encephalomyopathic with or without methylmalonic aciduria), 612073 (3)”

This gene has an homozygous variant (rs140963290) at 13-48528384 with a NON\_SYNONYMOUS\_CODING effect and a MODERATE impact.
This variant has a sift score of 0.00 and Polyphen2 score of 1.00

## Autosomal Recessive - Compound Heterozygous¶

1. Click on “1-Click” link at the top menu or go to http://mendel.medicina.ufmg.br/filter\_analysis/oneclick/
2. Add individual ‘NA12878 compound heterozygous’ to section SELECT VARIANTS FROM - INDIVIDUALS
3. Select Inheritance “Recessive Compound Heterozygous”
4. Click the “Submit” button.

Summary:
Number of Variants: 380
Number of Genes: 93
Number of Genes at OMIM: 14

Among the 14 candidate genes, there is the gene “HEXA” already known to be associated with “GM2-gangliosidosis, several forms, 272800 (3), Tay-Sachs disease, 272800 (3) and [Hex A pseudodeficiency], 272800 (3)”.

Here it’s is important to have some clinical skills to select the best candidate gene according to the phenotype and symptoms of each medical genetics case.

This gene contains two candidate variants.

The first heterozygous variant found (rs121907962) at 15-72647903 has a STOP\_GAINED effect and a HIGH impact.

The second heterozygous variant found (rs28941770) at 15-72645446 has a NON\_SYNONYMOUS\_CODING effect and a MODERATE impact.

> This variant has a sift score of 0.00 and a Polyphen2 score of 1.00

## Autosomal Dominant - Heterozygous¶

1. Click on “1-Click” link at the top menu or go to http://mendel.medicina.ufmg.br/filter\_analysis/oneclick/
2. Add individual ‘NA12878 dominant’ to section SELECT VARIANTS FROM - INDIVIDUALS
3. Select Inheritance “Dominant Heterozygous”
4. Click the “Submit” button.

Summary:
Number of Variants: 949
Number of Genes: 664
Number of Genes at OMIM: 148

Among the 148 genes at OMIM, you will find the gene “KCNA2” already known to be associated with “Epileptic encephalopathy, early infantile, 32, 616366 (3)”

This gene has an heterozygous variant (rs786205232) at 1-111146515 with a NON\_SYNONYMOUS\_CODING effect and a MODERATE impact.

This variant has a sift score of 0.00 and a Polyphen2 score of 0.98.

This would be a more difficult case to find the gene, but a list with 148 genes can be manually inspected by a clinician in the search for a good candidate.

## Dominant X-linked - Hemizygous¶

1. Click on “1-Click” link at the top menu or go to http://mendel.medicina.ufmg.br/filter\_analysis/oneclick/
2. Add individual ‘NA12878 xlinked’ to section SELECT VARIANTS FROM - INDIVIDUALS
3. Select Inheritance “X-linked recessive hemizygous”
4. Click the “Submit” button.

Summary:
Number of Variants: 2
Number of Genes: 2
Number of Genes at OMIM: 1

There is only one gene “F8” at OMIM. This gene is already known to be associated with “Hemophilia A, 306700 (3)”.

This gene has an hemyzigous variant at X-154250763 with a NON\_SYNONYMOUS\_CODING effect and a MODERATE impact.

This variant has a sift score of 0.00 and a Polyphen2 score of 1.00

### Table Of Contents

- Clinical Cases
  - Upload of Examples
  - Autosomal Recessive - Homozygous
  - Autosomal Recessive - Compound Heterozygous
  - Autosomal Dominant - Heterozygous
  - Dominant X-linked - Hemizygous

### Related Topics

- Documentation overview
  - Previous: Pynnotator
  - Next: Filter Analysis

### This Page

- Show Source

### Quick search

©2017, Raony Guimaraes.
|
Powered by Sphinx 1.5.5
& Alabaster 0.7.10
|
Page source
