# Supplementary material for: Mendel,MD: A user-friendly open-source web tool for analyzing WES and WGS in the diagnosis of patients with Mendelian disorders
Source: PLoS Comput Biol. 2017 Jun 8;13(6):e1005520. doi: 10.1371/journal.pcbi.1005520 (PMC5464533; doi:10.1371/journal.pcbi.1005520)
Supplement: S1 Code — Last version of the source-code of Mendel,MD. (ZIP) [file pcbi.1005520.s004.zip › mendelmd-master/mendelmd_source/static/docs/extension.html]

Extension — Mendel,MD 0.3 documentation


# Extension¶

Here we describe the steps to extend Mendel,MD and add a new options for annotation and filtering.

1. Create the necessary field to store this data on variants/models.py app.
2. Create the form to filter this data (Ex. slide, select box or input text field) on filter\_analysis/forms.py
3. Create the method that will filter this field on filter\_analysis/filter\_variants.py.
4. Add the field as a column in the filter\_analysis/templates of the variants template form.

### Related Topics

- Documentation overview
  - Previous: Filter Analysis

### This Page

- Show Source

### Quick search

©2017, Raony Guimaraes.
|
Powered by Sphinx 1.5.5
& Alabaster 0.7.10
|
Page source
