# Supplementary material for: Mendel,MD: A user-friendly open-source web tool for analyzing WES and WGS in the diagnosis of patients with Mendelian disorders
Source: PLoS Comput Biol. 2017 Jun 8;13(6):e1005520. doi: 10.1371/journal.pcbi.1005520 (PMC5464533; doi:10.1371/journal.pcbi.1005520)
Supplement: S1 Code — Last version of the source-code of Mendel,MD. (ZIP) [file pcbi.1005520.s004.zip › mendelmd-master/mendelmd_source/static/docs/filter_analysis.html]

Filter Analysis — Mendel,MD 0.3 documentation


# Filter Analysis¶

With this form we offer different options for filtering your variants based on the fields provided by different databases and tools such as Annovar, Snpeff, VEP, dbSNP, 1000Genomes, Exome Sequencing Project and some others.

## One Click¶

This method was implemented with the idea in mind that the doctors/researchers would always have an idea about the most possible inheritance type of the disease from the family in study. So after selecting beetween Recessive Homozygous, Recessive Heretozygous, Dominant or X-linked the use would get quickly a small list of variants that fits the selected inheritance mode.

We do this by defining the attributes according to the table describe above:

## Family Analysis¶

This method was created to allow the analysis of exomes from families.

### Formats¶

In order to fully understand this filtering process it’s usually required that you have some knowledge about some different formats, tools and scores of predictions and conservation.

Please before you start filtering your variants using Mendel,MD it’s recommended that you read the following links:

- VCF

http://1000genomes.org/wiki/Analysis/Variant Call Format/vcf-variant-call-format-version-41

- SnpEff

http://snpeff.sourceforge.net/SnpEff\_manual.html

- VEP

http://www.ensembl.org/info/genome/variation/predicted\_data.html#consequences
http://www.ensembl.org/info/docs/tools/vep/vep\_formats.html#output

### Quick Start¶

The method of filtering variants is based on different criterias that are grouped by tabs.

We have 5 main tabs that offer you filtering options: Individuals, VCF, Annovar, SnpEff and VEP.

Each one of this tabs have different options that enables you to filter variants from your individual.

## Individuals Options¶

On this section you have two columns “Select Variants From” and “Exclude Variants From”. The first column enables you to include individuals that you uploaded, snps from dbsnp138 and genes symbols from refseq that you want to see in your results. The second column is where you can choose also individuals, snps and genes that you want to exclude from your results.

## VCF Options¶

This Desction let you select fields from your VCF File(s).

- Mutation Types

With this field you can choose beetween mohozygous or heterzygous variants.

Ex. Homozygous (Ex. 1/1, 1/2, 2/3 and etc) => for recessive models on inheritance
:   Heterozygous (Ex. 0/1, 0/2, 0/2 and etc) => for dominant models on inheritance

Usage: This is usefull if you want you want to search for recessive and dominant models of inheritance.

- Chr

With this field you can choose to see only variants from a single chromossome.
Ex. X, Y, 1, 2 ... 22

-Pos
This option let you choose variants in certain regions of a chromossome based in a string.

Ex. Chr 1: Pos:12345-12360

(this will return all variants beetween the positions 12345-12360 of Chromossome 1)

- Filter Type

With this option you can select variants based on the FILTER column of your VCF.

Ex. PASS, LowQual, repeat, SnpCluster
Usage: You can select variants that are only PASS in you VCF file(s)

- dbSNP Build

With this option you can choose only variants after a certain build of last version of dbSNP
Ex. >= 130 (will get only variants after dbsnp 130)

NOTE: dbSNP 129 is generally regarded as the last “clean” dbSNP without “contamination” from 1000 Genomes Project and other large-scale next-generation sequencing projects. Many published papers utilize dbSNP129 only.
Source: http://www.openbioinformatics.org/annovar/annovar\_filter.html#dbsnp

- Read Depth

This option should be choosen based on the mean coverage of the individuals (exomes, genomes) that you selected under the previous section “Individuals”
Ex. >= 30 (This will select only variants with more than 29 reads of coverage and can be used for filtering an exome with 80X of coverage)

- Qual

QUAL phred-scaled quality score for the assertion made in ALT. i.e. -10log\_10 prob(call in ALT is wrong). If ALT is ”.” (no variant) then this is -10log\_10 p(variant), and if ALT is not ”.” this is -10log\_10 p(no variant). High QUAL scores indicate high confidence calls. Although traditionally people use integer phred scores, this field is permitted to be a floating point to enable higher resolution for low confidence calls if desired. If unknown, the missing value should be specified. (Numeric)
This option let you choose beetwen the quality score of the variant based on the value provided by VCF.
Ex. >= 50

Source: www.1000genomes.org/wiki/Analysis/Variant Call Format/vcf-variant-call-format-version-41

- Records per gene

This option let you choose how many different variants per gene you want to see in your results
Ex. <= 2 (will show only variants in genes that have less than or equal 2 variants per gene for each individual)

- Show only records in genes common to all the individuals selected

With this option you can search for variants in genes that are common to all individuals selected.

## Annovar Options¶

List of fields that can be filtered in Mendel,MD: all from annovar, snpeff and VEP.

Annovar Fields

Source: http://www.openbioinformatics.org/annovar/annovar\_db.html

- Func.refGene
- Gene.refGene
- ExonicFunc.refGene
- AAChange.refGene
- phastConsElements46way
- genomicSuperDups
- esp6500si\_all
- 1000g2012apr\_all
- LJB2\_SIFT
- LJB2\_PolyPhen2\_HDIV
- LJB2\_PP2\_HDIV\_Pred
- LJB2\_PolyPhen2\_HVAR
- LJB2\_PolyPhen2\_HVAR\_Pred
- LJB2\_LRT
- LJB2\_LRT\_Pred
- LJB2\_MutationTaster
- LJB2\_MutationTaster\_Pred
- LJB\_MutationAssessor
- LJB\_MutationAssessor\_Pred
- LJB2\_FATHMM
- LJB2\_GERP++
- LJB2\_PhyloP
- LJB2\_SiPhy
- cosmic65
- avsift

# Description of the Fields¶

## Func refGene¶

With this option you can choose the region of your variant:
Ex. [‘splicing’, ‘UTR5’, ‘ncRNA\_exonic’, ‘intergenic’, ‘intronic’, ‘UTR3’, ‘exonic’, ‘upstream’, ‘ncRNA\_intronic’]

## Gene.refGene¶

Description: Known human protein-coding and non-protein-coding genes taken from the NCBI RNA reference sequences collection (RefSeq)

Usage:
With this option you can choose the gene Simbol of a variant.
Ex. SUCLA2

Source: http://www.ncbi.nlm.nih.gov/refseq/

## ExonicFunc.refGene¶

Description: Exonic variant function (non-synonymous, synonymous, etc)

Usage:[‘frameshift\_insertion’, ‘frameshift\_deletion’, ‘frameshift\_block\_substitution’, ‘stopgain’, ‘stoploss’, ‘nonframeshift\_insertion’, ‘nonframeshift\_deletion’, ‘nonframeshift\_block\_substitution’, ‘nonsynonymous\_SNV’, ‘synonymous\_SNV’, ‘unknown’]

## AAChange.refGene¶

Description: Amino acid changes

## phastConsElements46way¶

Description: Conserved elements produced by the phastCons program based on a whole-genome alignment of vertebrates.
There is no specific recommended cutoff for highly conserved elements.

PhastCons (which has been used in previous Conservation tracks) is a hidden Markov model-based method that estimates the probability that each nucleotide belongs to a conserved element, based on the multiple alignment. It considers not just each individual alignment column, but also its flanking columns. By contrast, phyloP separately measures conservation at individual columns, ignoring the effects of their neighbors. As a consequence, the phyloP plots have a less smooth appearance than the phastCons plots, with more “texture” at individual sites. The two methods have different strengths and weaknesses. PhastCons is sensitive to “runs” of conserved sites, and is therefore effective for picking out conserved elements. PhyloP, on the other hand, is more appropriate for evaluating signatures of selection at particular nucleotides or classes of nucleotides (e.g., third codon positions, or first positions of miRNA target sites).

Usage: With this option you can select only varinats that are conserved among 46 vertebrate species

Sources:
http://compgen.bscb.cornell.edu/phast/phastCons-HOWTO.html
http://genome.ucsc.edu/cgi-bin/hgTrackUi?db=hg19&g=cons46way

## genomicSuperDups¶

Description: Segmental duplications in genome.
Duplications of >1000 Bases of Non-RepeatMasked Sequence (>90 percent similar)

Regions detected as putative genomic duplications. For overlap with each of those regions another chromosome and location are reported.

Usage:
With this option you can exclude variants in regions of Segmental duplication

## esp6500si\_all¶

Description: alternative allele frequency in all subjects in the NHLBI-ESP project with 6500 exomes.
Usage:
With this option you can select only variants with a certain frequency in this databse.
There is also an option to exclude all variants seen in this database.
Source:
http://evs.gs.washington.edu

## 1000g2012apr\_all¶

Description: alternative allele frequency data in 1000 Genomes Project (1000g2012apr)
Usage:
With this option you can select only variants with a certain frequency in this databse.
There is also an option to exclude all variants seen in this database.
Source:
http://1000genomes.org

## LJB2\_SIFT¶

Description:
SIFT predicts whether an amino acid substitution affects protein function. SIFT prediction is based on the degree of conservation of amino acid residues in sequence alignments derived from closely related sequences, collected through PSI-BLAST. SIFT can be applied to naturally occurring nonsynonymous polymorphisms or laboratory-induced missense mutations.

Usage:
Ex.
<= 0.05

With this option you can select only variants predicted as “damaging” by LJB2\_SIFT.
You can also exclude all variants that does not have a sift score from your results.
obs: remember that indels and frameshifts doesn’t have sift scores.

Source: http://sift.jcvi.org/

Kumar P, Henikoff S, Ng PC. Predicting the effects of coding non-synonymous variants on protein function using the SIFT algorithm. Nat Protoc. 2009;4(7):1073-81. PubMed PDF

## LJB2\_PolyPhen2\_HDIV¶

Description: Whole-exome PolyPhen scores built on HumanDiv database (for complex phenotypes)

PolyPhen-2 (Polymorphism Phenotyping v2) is a tool which predicts possible impact of an amino acid substitution on the structure and function of a human protein using straightforward physical and comparative considerations. Please, use the form below to submit your query.

Usage:

Source:
http://genetics.bwh.harvard.edu/pph2/bgi.shtml

Adzhubei IA, Schmidt S, Peshkin L, Ramensky VE, Gerasimova A, Bork P, Kondrashov AS, Sunyaev SR. 2010. A method and server for predicting damaging missense mutations. Nature Methods 7: 248–249.

## LJB2\_PP2\_HDIV\_Pred¶

Description
Usage:
(‘D’, ‘probably damaging’),
(‘P’, ‘possibly damaging’),
(‘B’, ‘benign’)

## LJB2\_PolyPhen2\_HVAR¶

Description: whole-exome PolyPhen version 2 scores built on HumanVar database (for Mendelian phenotypes)

## LJB2\_PolyPhen2\_HVAR\_Pred¶

Description
Usage:
(‘D’, ‘probably damaging’),
(‘P’, ‘possibly damaging’),
(‘B’, ‘benign’)

## LJB2\_LRT¶

Chun S, Fay JC. 2009. Identification of deleterious mutations within three human genomes. Genome Research 19: 1553 –1561.
Cooper

## LJB2\_LRT\_Pred¶

Description
Usage:
(‘D’, ‘probably damaging’),
(‘P’, ‘possibly damaging’),
(‘B’, ‘benign’)

## LJB2\_MutationTaster¶

Schwarz JM, Rodelsperger C, Schuelke M, Seelow D. 2010. MutationTaster evaluates disease-causing potential of sequence alterations. Nature Methods 7: 575–576.

## LJB2\_MutationTaster\_Pred¶

Description
Usage:
(‘D’, ‘probably damaging’),
(‘P’, ‘possibly damaging’),
(‘B’, ‘benign’)

## LJB\_MutationAssessor¶

http://mutationassessor.org/

References

Reva B, Antipin Y, Sander C. 2011. Predicting the functional impact of protein mutations: Application to cancer genomics. Nucleic Acids Research 39: e118

## LJB\_MutationAssessor\_Pred¶

Description
Usage:
(‘D’, ‘probably damaging’),
(‘P’, ‘possibly damaging’),
(‘B’, ‘benign’)

## LJB2\_FATHMM¶

http://fathmm.biocompute.org.uk/

Shihab HA, Gough J, Cooper DN, Stenson PD, Barker GLA, Edwards KJ, Day INM, Gaunt TR. 2013. Predicting the functional, molecular, and phenotypic consequences of amino acid substitutions using hidden Markov models. Human Mutation 34: 57–65.

## LJB2\_GERP++¶

http://mendel.stanford.edu/SidowLab/downloads/gerp/

Davydov EV, Goode DL, Sirota M, Cooper GM, Sidow A, Batzoglou S. 2010. Identifying a high fraction of the human genome to be under selective constraint using GERP++. PLoS Comput Biol 6: e1001025.

## LJB2\_PhyloP¶

Description Compute conservation or acceleration p-values based on an alignment and
:   a model of neutral evolution.

Siepel A, Pollard KS, and Haussler D. 2006. New methods for detecting lineage-specific selection. Proceedings of the 10th International Conference on Research in Computational Molecular Biology (RECOMB 2006): 190-205.

## LJB2\_SiPhy¶

http://www.broadinstitute.org/mammals/2x/siphy\_hg19/

Garber M, Guttman M, Clamp M, Zody MC, Friedman N, Xie X. 2009. Identifying novel constrained elements by exploiting biased substitution patterns. Bioinformatics 25: i54–i62.

Lindblad-Toh K, Garber M, Zuk O, Lin MF, Parker BJ, Washietl S, Kheradpour P, Ernst J, Jordan G, Mauceli E, Ward LD, Lowe CB, et al. 2011. A high-resolution map of human evolutionary constraint using 29 mammals. Nature 478: 476–482

## cosmic65¶

## avsift¶

> func\_refgene\_options = [‘splicing’, ‘UTR5’, ‘ncRNA\_exonic’, ‘intergenic’, ‘intronic’, ‘UTR3’, ‘exonic’, ‘upstream’, ‘ncRNA\_intronic’]

Source:

dbNSFP: a lightweight database of human nonsynonymous SNPs and their functional predictions.
Liu X, Jian X, Boerwinkle E.
http://www.ncbi.nlm.nih.gov/pubmed/21520341

dbNSFP v2.0: a database of human non-synonymous SNVs and their functional predictions and annotations.
Liu X, Jian X, Boerwinkle E.
http://www.ncbi.nlm.nih.gov/pubmed/23843252

### Annotations from SNPEFF¶

- SNPEFF\_AMINO\_ACID\_CHANGE
- SNPEFF\_CODON\_CHANGE
- SNPEFF\_EFFECT
- SNPEFF\_EXON\_ID
- SNPEFF\_FUNCTIONAL\_CLASS
- SNPEFF\_GENE\_BIOTYPE
- SNPEFF\_GENE\_NAME
- SNPEFF\_IMPACT
- SNPEFF\_TRANSCRIPT\_ID

SNPEFF\_AMINO\_ACID\_CHANGE

Old/New amino acid for the highest-impact effect resulting from the current variant (in HGVS styl
e)

SNPEFF\_CODON\_CHANGE

Old/New codon for the highest-impact effect resulting from the current variant

SNPEFF\_EFFECT

The highest-impact effect resulting from the current variant (or one of the highest-impact effects, if there is a tie)

SNPEFF\_EXON\_ID

Exon ID for the highest-impact effect resulting from the current variant

SNPEFF\_FUNCTIONAL\_CLASS

Functional class of the highest-impact effect resulting from the current variant: [NONE, SILENT, M
ISSENSE, NONSENSE]

SNPEFF\_GENE\_BIOTYPE

Gene biotype for the highest-impact effect resulting from the current variant

SNPEFF\_GENE\_NAME

Gene name for the highest-impact effect resulting from the current variant

SNPEFF\_IMPACT

Impact of the highest-impact effect resulting from the current variant [MODIFIER, LOW, MODERATE, HIGH]

SNPEFF\_TRANSCRIPT\_ID

Transcript ID for the highest-impact effect resulting from the current variant

Sources:
http://snpeff.sourceforge.net/SnpEff\_manual.html
http://snpeff.sourceforge.net/SnpEff\_manual.html#How\_is\_impact\_categorized?\_%28VCF\_output%29

### VEP Options¶

List of fields:

- Allele
- Gene
- Feature
- Feature\_type
- Consequence
- cDNA\_position
- CDS\_position
- Protein\_position
- Amino\_acids
- Codons
- Existing\_variation
- DISTANCE
- SIFT
- PolyPhen
- CELL\_TYPE
- Condel

http://www.ensembl.org/info/docs/tools/vep/vep\_formats.html#output
Allele - the variant allele used to calculate the consequence
Gene - Ensembl stable ID of affected gene
Feature - Ensembl stable ID of feature
Feature\_type - type of feature. Currently one of Transcript, RegulatoryFeature, MotifFeature.
Consequence http://www.ensembl.org/info/genome/variation/predicted\_data.html#consequences
cDNA\_position
CDS\_position
Protein\_position
Amino\_acids
Codons
Existing\_variation
DISTANCE
CELL\_TYPE

## SIFT¶

SIFT predicts whether an amino acid substitution is likely to affect protein function based on sequence homology and the physico-chemical similarity between the alternate amino acids. The data we provide for each amino acid substitution is a score and a qualitative prediction (either ‘tolerated’ or ‘deleterious’). The score is the normalized probability that the amino acid change is tolerated so scores nearer 0 are more likely to be deleterious. The qualitative prediction is derived from this score such that substitutions with a score < 0.05 are called ‘deleterious’ and all others are called ‘tolerated’.

We ran SIFT version 5.0.2 following the instructions from the authors and used SIFT to choose homologous proteins rather than supplying them ourselves. We used all protein sequences available from UniRef90 (release 2012\_11) as the protein database.

## PolyPhen-2¶

PolyPhen-2 predicts the effect of an amino acid substitution on the structure and function of a protein using sequence homology, Pfam annotations, 3D structures from PDB where available, and a number of other databases and tools (including DSSP, ncoils etc.). As with SIFT, for each amino acid substitution where we have been able to calculate a prediction, we provide both a qualitative prediction (one of ‘probably damaging’, ‘possibly damaging’, ‘benign’ or ‘unknown’) and a score. The PolyPhen score represents the probability that a substitution is damaging, so values nearer 1 are more confidently predicted to be deleterious (note that this the opposite to SIFT). The qualitative prediction is based on the False Positive Rate of the classifier model used to make the predictions.

We ran PolyPhen-2 version 2.2.2 (available here) and again we followed all instructions from the authors, and used the UniProtKB UniRef100 (release 2011\_12) non-redundant protein set as the protein database, which was downloaded, along with PDB structures, and annotations from Pfam and DSSP(snapshot 03-Jan-2012) in February 2012. When computing the predictions we store results for the classifier models trained on the HumDiv and HumVar datasets. Both result sets are available through the variation API which defaults to HumVar if no selection is made. (Please refer to the PolyPhen website or publications for more details of the classification system).

## Condel¶

Condel is a general method for calculating a consensus prediction from the output of tools designed to predict the effect of an amino acid substitution. It does so by calculating a weighted average score (WAS) of the scores of each component method. The Condel authors provided us with a version specialised for finding a consensus between SIFT and PolyPhen and we integrated this into a Variation Effect Predictor (VEP) plugin. Tests run by the authors on the HumVar dataset (a test set curated by the PolyPhen team), show that Condel can improve both the sensitivity and specificity of predictions compared to either SIFT or PolyPhen used alone (please contact the authors for details). The Condel score, along with a qualitative prediction (one of ‘neutral’ or ‘deleterious’), are available in the VEP plugin. The Condel score is the consensus probability that a substitution is deleterious, so values nearer 1 are predicted with greater confidence to affect protein function.

Sources: http://www.ensembl.org/info/genome/variation/predicted\_data.html#consequences

### Table Of Contents

- Filter Analysis
  - One Click
  - Family Analysis
    - Formats
    - Quick Start
  - Individuals Options
  - VCF Options
  - Annovar Options
- Description of the Fields
  - Func refGene
  - Gene.refGene
  - ExonicFunc.refGene
  - AAChange.refGene
  - phastConsElements46way
  - genomicSuperDups
  - esp6500si\_all
  - 1000g2012apr\_all
  - LJB2\_SIFT
  - LJB2\_PolyPhen2\_HDIV
  - LJB2\_PP2\_HDIV\_Pred
  - LJB2\_PolyPhen2\_HVAR
  - LJB2\_PolyPhen2\_HVAR\_Pred
  - LJB2\_LRT
  - LJB2\_LRT\_Pred
  - LJB2\_MutationTaster
  - LJB2\_MutationTaster\_Pred
  - LJB\_MutationAssessor
  - LJB\_MutationAssessor\_Pred
  - LJB2\_FATHMM
  - LJB2\_GERP++
  - LJB2\_PhyloP
  - LJB2\_SiPhy
  - cosmic65
  - avsift
    - Annotations from SNPEFF
    - VEP Options
  - SIFT
  - PolyPhen-2
  - Condel

### Related Topics

- Documentation overview
  - Previous: Clinical Cases
  - Next: Extension

### This Page

- Show Source

### Quick search

©2017, Raony Guimaraes.
|
Powered by Sphinx 1.5.5
& Alabaster 0.7.10
|
Page source
