# Supplementary material for: Mendel,MD: A user-friendly open-source web tool for analyzing WES and WGS in the diagnosis of patients with Mendelian disorders
Source: PLoS Comput Biol. 2017 Jun 8;13(6):e1005520. doi: 10.1371/journal.pcbi.1005520 (PMC5464533; doi:10.1371/journal.pcbi.1005520)
Supplement: S1 Code — Last version of the source-code of Mendel,MD. (ZIP) [file pcbi.1005520.s004.zip › mendelmd-master/mendelmd_source/static/docs/index.html]

Welcome to Mendel,MD’s documentation! — Mendel,MD 0.3 documentation


# Welcome to Mendel,MD’s documentation!¶

Contents:

- Mendel,MD
- Requirements
- Other Libraries Needed
- OMIM Data
- Docker
- Manual Installation
- Installing PostgreSQL Database
- Installation on Ubuntu 16.04 LTS (tested)
- Installing the Pynnotator
- Installing Mendel,MD
- Creating a superuser
- Importing Data
- Upload your VCFs using the web interface
- Start the annotation
- Deployment on RedHat/CentOS 7
- Pynnotator
- Libraries
- Databases
- Features
- Requirements
- Installation
- Test
- Clinical Cases
  - Upload of Examples
  - Autosomal Recessive - Homozygous
  - Autosomal Recessive - Compound Heterozygous
  - Autosomal Dominant - Heterozygous
  - Dominant X-linked - Hemizygous
- Filter Analysis
  - One Click
  - Family Analysis
  - Individuals Options
  - VCF Options
  - Annovar Options
- Description of the Fields
  - Func refGene
  - Gene.refGene
  - ExonicFunc.refGene
  - AAChange.refGene
  - phastConsElements46way
  - genomicSuperDups
  - esp6500si\_all
  - 1000g2012apr\_all
  - LJB2\_SIFT
  - LJB2\_PolyPhen2\_HDIV
  - LJB2\_PP2\_HDIV\_Pred
  - LJB2\_PolyPhen2\_HVAR
  - LJB2\_PolyPhen2\_HVAR\_Pred
  - LJB2\_LRT
  - LJB2\_LRT\_Pred
  - LJB2\_MutationTaster
  - LJB2\_MutationTaster\_Pred
  - LJB\_MutationAssessor
  - LJB\_MutationAssessor\_Pred
  - LJB2\_FATHMM
  - LJB2\_GERP++
  - LJB2\_PhyloP
  - LJB2\_SiPhy
  - cosmic65
  - avsift
  - SIFT
  - PolyPhen-2
  - Condel
- Extension

# Indices and tables¶

- Index
- Module Index
- Search Page

### Table Of Contents

- Welcome to Mendel,MD’s documentation!
- Indices and tables

### Related Topics

- Documentation overview
  - Next: Mendel,MD

### This Page

- Show Source

### Quick search

©2017, Raony Guimaraes.
|
Powered by Sphinx 1.5.5
& Alabaster 0.7.10
|
Page source
