# Supplementary material for: Mendel,MD: A user-friendly open-source web tool for analyzing WES and WGS in the diagnosis of patients with Mendelian disorders
Source: PLoS Comput Biol. 2017 Jun 8;13(6):e1005520. doi: 10.1371/journal.pcbi.1005520 (PMC5464533; doi:10.1371/journal.pcbi.1005520)
Supplement: S1 Code — Last version of the source-code of Mendel,MD. (ZIP) [file pcbi.1005520.s004.zip › mendelmd-master/mendelmd_source/static/docs/installation.html]

Mendel,MD — Mendel,MD 0.3 documentation


# Mendel,MD¶

An online tool for annotating, filtering and diagnosing Humans (Exome
and Genome) with Mendelian Disorders.

# Requirements¶

- Python 3.4+
- Perl 5.8+
- Java 1.8
- Ubuntu LTS 16.04

# Other Libraries Needed¶

- pynnotator

# OMIM Data¶

You need to register an account at OMIM: http://omim.org/downloads and
submit a download request to get a file named “morbidmap”.

After obtaining this file you need to put it inside this folder:
“mendelmd\_source/data/omim/”.

# Docker¶

Now it’s possible to install and run Mendel,MD with a single command:

```
docker-compose up
```

This command will create a container, download all the required tools and datasets and run the webserver.

This should take a few hours to complete for the first time.

# Manual Installation¶

# Installing PostgreSQL Database¶

```
sudo apt-get install libpq-dev postgresql
sudo -i -u postgres
createuser --interactive
createdb mendelmd
cp mendelmd/local_settings.sample.py mendelmd/local_settings.py
```

# Installation on Ubuntu 16.04 LTS (tested)¶

```
sudo apt-get install gcc git python3-dev virtualenvwrapper zip zlibc zlib1g zlib1g-dev build-essential libssl-dev libffi-dev python-dev python3-dev python3-venv

python3 -m venv mendelmdenv
source mendelmdenv/bin/activate

git clone https://github.com/raonyguimaraes/mendelmd.git
cd mendelmd/mendelmd_source
```

# Installing the Pynnotator¶

```
pip install cython
pip install pynnotator
pynnotator install
```

This will take a long time since it will download around 26GB of data.
Go grab a coffee and read a paper.

# Installing Mendel,MD¶

```
pip install -r requirements.stable.txt

python manage.py migrate auth
python manage.py migrate
python manage.py populate
python manage.py runserver
```

And now you should have Mendel,MD running on address
http://127.0.0.1:8000/

# Creating a superuser¶

```
python manage.py createsuperuser
```

# Importing Data¶

```
python manage.py populate
```

# Upload your VCFs using the web interface¶

# Start the annotation¶

```
python manage.py celery worker
```

# Deployment on RedHat/CentOS 7¶

https://www.digitalocean.com/community/tutorials/how-to-serve-django-applications-with-apache-and-mod\_wsgi-on-centos-7

```
sudo yum -y install wget
wget https://data.omim.org/downloads/ADDYOURKEY/morbidmap.txt -O /tmp/morbidmap.txt
wget https://raw.github.com/raonyguimaraes/mendelmd/master/scripts/deployment_centos7_redhat7.sh
bash deployment_centos7_redhat7.sh
```

### Table Of Contents

- Mendel,MD
- Requirements
- Other Libraries Needed
- OMIM Data
- Docker
- Manual Installation
- Installing PostgreSQL Database
- Installation on Ubuntu 16.04 LTS (tested)
- Installing the Pynnotator
- Installing Mendel,MD
- Creating a superuser
- Importing Data
- Upload your VCFs using the web interface
- Start the annotation
- Deployment on RedHat/CentOS 7

### Related Topics

- Documentation overview
  - Previous: Welcome to Mendel,MD’s documentation!
  - Next: Pynnotator

### This Page

- Show Source

### Quick search

©2017, Raony Guimaraes.
|
Powered by Sphinx 1.5.5
& Alabaster 0.7.10
|
Page source
