# Supplementary material for: Mendel,MD: A user-friendly open-source web tool for analyzing WES and WGS in the diagnosis of patients with Mendelian disorders
Source: PLoS Comput Biol. 2017 Jun 8;13(6):e1005520. doi: 10.1371/journal.pcbi.1005520 (PMC5464533; doi:10.1371/journal.pcbi.1005520)
Supplement: S1 Code — Last version of the source-code of Mendel,MD. (ZIP) [file pcbi.1005520.s004.zip › mendelmd-master/mendelmd_source/static/jQuery-File-Upload-9.5.8/angularjs.html]

jQuery File Upload Demo - AngularJS version


jQuery File Upload

- Download
- Source Code
- Documentation
- © Sebastian Tschan

# jQuery File Upload Demo

## AngularJS version

- Basic
- Basic Plus
- Basic Plus UI
- AngularJS
- jQuery UI

  
> File Upload widget with multiple file selection, drag&drop support, progress bars, validation and preview images, audio and video for AngularJS.  
> Supports cross-domain, chunked and resumable file uploads and client-side image resizing.  
> Works with any server-side platform (PHP, Python, Ruby on Rails, Java, Node.js, Go etc.) that supports standard HTML form file uploads.

  


Add files...


Start upload


Cancel upload

|  |  |  |  |
| --- | --- | --- | --- |
|  | {{file.name}} {{file.name}} {{file.name}} **{{file.error}}** | {{file.size \| formatFileSize}} | Start   Cancel   Delete |

  

### Demo Notes

- The maximum file size for uploads in this demo is **5 MB** (default file size is unlimited).
- Only image files (**JPG, GIF, PNG**) are allowed in this demo (by default there is no file type restriction).
- Uploaded files will be deleted automatically after **5 minutes** (demo setting).
- You can **drag & drop** files from your desktop on this webpage (see Browser support).
- Please refer to the project website and documentation for more information.
- Built with Twitter's Bootstrap CSS framework and Icons from Glyphicons.

‹
›
×
