# Supplementary material for: Mendel,MD: A user-friendly open-source web tool for analyzing WES and WGS in the diagnosis of patients with Mendelian disorders
Source: PLoS Comput Biol. 2017 Jun 8;13(6):e1005520. doi: 10.1371/journal.pcbi.1005520 (PMC5464533; doi:10.1371/journal.pcbi.1005520)
Supplement: S1 Code — Last version of the source-code of Mendel,MD. (ZIP) [file pcbi.1005520.s004.zip › mendelmd-master/mendelmd_source/static/jQuery-File-Upload-9.5.8/jquery-ui.html]

jQuery File Upload Demo - jQuery UI version


- ### jQuery File Upload
- Download
- Source Code
- Documentation
- © blueimp.net

# jQuery File Upload Demo

## jQuery UI version

Theme:

Black Tie
Blitzer
Cupertino
Dark Hive
Dot Luv
Eggplant
Excite Bike
Flick
Hot sneaks
Humanity
Le Frog
Mint Choc
Overcast
Pepper Grinder
Redmond
Smoothness
South Street
Start
Sunny
Swanky Purse
Trontastic
UI Darkness
UI Lightness
Vader

- Basic
- Basic Plus
- Basic Plus UI
- AngularJS
- jQuery UI

> File Upload widget with multiple file selection, drag&drop support, progress bars, validation and preview images, audio and video for jQuery UI.  
> Supports cross-domain, chunked and resumable file uploads and client-side image resizing.  
> Works with any server-side platform (PHP, Python, Ruby on Rails, Java, Node.js, Go etc.) that supports standard HTML form file uploads.


Add files...

Start upload
Cancel upload
Delete

  

### Demo Notes

- The maximum file size for uploads in this demo is **5 MB** (default file size is unlimited).
- Only image files (**JPG, GIF, PNG**) are allowed in this demo (by default there is no file type restriction).
- Uploaded files will be deleted automatically after **5 minutes** (demo setting).
- You can **drag & drop** files from your desktop on this webpage (see Browser support).
- Please refer to the project website and documentation for more information.
- Built with jQuery UI.

‹
›
×
