# Supplementary material for: Mendel,MD: A user-friendly open-source web tool for analyzing WES and WGS in the diagnosis of patients with Mendelian disorders
Source: PLoS Comput Biol. 2017 Jun 8;13(6):e1005520. doi: 10.1371/journal.pcbi.1005520 (PMC5464533; doi:10.1371/journal.pcbi.1005520)
Supplement: S1 Code — Last version of the source-code of Mendel,MD. (ZIP) [file pcbi.1005520.s004.zip › mendelmd-master/mendelmd_source/templates/account/email.html]

{% extends "account/base.html" %}
{% load i18n %}
{% block head\_title %}{% trans "Account" %}{% endblock %}
{% block content %}

# {% trans "E-mail Addresses" %}

{% if user.emailaddress\_set.all %}

{% trans 'The following e-mail addresses are associated with your account:' %}

{% csrf\_token %}
{% for emailaddress in user.emailaddress\_set.all %}

{{ emailaddress.email }}
{% if emailaddress.verified %}
{% trans "Verified" %}
{% else %}
{% trans "Unverified" %}
{% endif %}
{% if emailaddress.primary %}{% trans "Primary" %}{% endif %}

{% endfor %}

{% trans 'Make Primary' %}
{% trans 'Re-send Verification' %}
{% trans 'Remove' %}

{% else %}

**{% trans 'Warning:'%}** {% trans "You currently do not have any e-mail address set up. You should really add an e-mail address so you can receive notifications, reset your password, etc." %}

{% endif %}

## {% trans "Add E-mail Address" %}

{% csrf\_token %}
{{ form.as\_p}}
{% trans "Add E-mail" %}
{% endblock %}
{% block extra\_body %}
{% endblock %}
