# Supplementary material for: Mendel,MD: A user-friendly open-source web tool for analyzing WES and WGS in the diagnosis of patients with Mendelian disorders
Source: PLoS Comput Biol. 2017 Jun 8;13(6):e1005520. doi: 10.1371/journal.pcbi.1005520 (PMC5464533; doi:10.1371/journal.pcbi.1005520)
Supplement: S1 Code — Last version of the source-code of Mendel,MD. (ZIP) [file pcbi.1005520.s004.zip › mendelmd-master/mendelmd_source/templates/account/email_confirm.html]

{% extends "account/base.html" %}
{% load i18n %}
{% load account %}
{% block head\_title %}{% trans "Confirm E-mail Address" %}{% endblock %}
{% block content %}

# {% trans "Confirm E-mail Address" %}

{% if confirmation %}
{% user\_display confirmation.email\_address.user as user\_display %}

{% blocktrans with confirmation.email\_address.email as email %}Please confirm that {{ email }} is an e-mail address for user {{ user\_display }}.{% endblocktrans %}

{% csrf\_token %}
{% trans 'Confirm' %}
{% else %}
{% url 'account\_email' as email\_url %}

{% blocktrans %}This e-mail confirmation link expired or is invalid. Please issue a new e-mail confirmation request.{% endblocktrans %}

{% endif %}
{% endblock %}
