# Supplementary material for: Mendel,MD: A user-friendly open-source web tool for analyzing WES and WGS in the diagnosis of patients with Mendelian disorders
Source: PLoS Comput Biol. 2017 Jun 8;13(6):e1005520. doi: 10.1371/journal.pcbi.1005520 (PMC5464533; doi:10.1371/journal.pcbi.1005520)
Supplement: S1 Code — Last version of the source-code of Mendel,MD. (ZIP) [file pcbi.1005520.s004.zip › mendelmd-master/mendelmd_source/templates/account/login.html]

{% extends "account/base.html" %}
{% load i18n %}
{% load account %}
{% block head\_title %}{% trans "Sign In" %}{% endblock %}
{% block content %}

# {% trans "Sign In" %}

{% if socialaccount.providers %}

{% blocktrans with site.name as site\_name %}Please sign in with one
of your existing third party accounts. Or, sign up
for a {{site\_name}} account and sign in below:{% endblocktrans %}

{% include "socialaccount/snippets/provider\_list.html" with process="login" %}

{% trans 'or' %}

{% include "socialaccount/snippets/login\_extra.html" %}
{% else %}

{% blocktrans %}If you have not created an account yet, then please
sign up first.{% endblocktrans %}

{% endif %}
{% csrf\_token %}
{{ form.as\_p }}
{% if redirect\_field\_value %}
{% endif %}
{% trans "Forgot Password?" %}
{% trans "Sign In" %}
{% endblock %}
