# Supplementary material for: Mendel,MD: A user-friendly open-source web tool for analyzing WES and WGS in the diagnosis of patients with Mendelian disorders
Source: PLoS Comput Biol. 2017 Jun 8;13(6):e1005520. doi: 10.1371/journal.pcbi.1005520 (PMC5464533; doi:10.1371/journal.pcbi.1005520)
Supplement: S1 Code — Last version of the source-code of Mendel,MD. (ZIP) [file pcbi.1005520.s004.zip › mendelmd-master/mendelmd_source/templates/account/password_reset.html]

{% extends "account/base.html" %}
{% load i18n %}
{% load account %}
{% block head\_title %}{% trans "Password Reset" %}{% endblock %}
{% block content %}

# {% trans "Password Reset" %}

{% if user.is\_authenticated %}
{% include "account/snippets/already\_logged\_in.html" %}
{% endif %}

{% trans "Forgotten your password? Enter your e-mail address below, and we'll send you an e-mail allowing you to reset it." %}

{% csrf\_token %}
{{ form.as\_p }}

{% blocktrans %}Please contact us if you have any trouble resetting your password.{% endblocktrans %}

{% endblock %}
{% block extra\_body %}
{% endblock %}
