# Supplementary material for: Mendel,MD: A user-friendly open-source web tool for analyzing WES and WGS in the diagnosis of patients with Mendelian disorders
Source: PLoS Comput Biol. 2017 Jun 8;13(6):e1005520. doi: 10.1371/journal.pcbi.1005520 (PMC5464533; doi:10.1371/journal.pcbi.1005520)
Supplement: S1 Code — Last version of the source-code of Mendel,MD. (ZIP) [file pcbi.1005520.s004.zip › mendelmd-master/mendelmd_source/templates/account/snippets/already_logged_in.html]

{% load i18n %}
{% load account %}
{% user\_display user as user\_display %}

**{% trans "Note" %}:** {% blocktrans %}you are already logged in as {{ user\_display }}.{% endblocktrans %}
