# Supplementary material for: Mendel,MD: A user-friendly open-source web tool for analyzing WES and WGS in the diagnosis of patients with Mendelian disorders
Source: PLoS Comput Biol. 2017 Jun 8;13(6):e1005520. doi: 10.1371/journal.pcbi.1005520 (PMC5464533; doi:10.1371/journal.pcbi.1005520)
Supplement: S1 Code — Last version of the source-code of Mendel,MD. (ZIP) [file pcbi.1005520.s004.zip › mendelmd-master/mendelmd_source/templates/account/verification_sent.html]

{% extends "account/base.html" %}
{% load i18n %}
{% block head\_title %}{% trans "Verify Your E-mail Address" %}{% endblock %}
{% block content %}

# {% trans "Verify Your E-mail Address" %}

{% blocktrans %}We have sent an e-mail to you for verification. Follow the link provided to finalize the signup process. Please contact us if you do not receive it within a few minutes.{% endblocktrans %}

{% endblock %}
