# Supplementary material for: Mendel,MD: A user-friendly open-source web tool for analyzing WES and WGS in the diagnosis of patients with Mendelian disorders
Source: PLoS Comput Biol. 2017 Jun 8;13(6):e1005520. doi: 10.1371/journal.pcbi.1005520 (PMC5464533; doi:10.1371/journal.pcbi.1005520)
Supplement: S1 Code — Last version of the source-code of Mendel,MD. (ZIP) [file pcbi.1005520.s004.zip › mendelmd-master/mendelmd_source/templates/base.html]

{% load static i18n %}


Mendel,MD - Cloud Based Genome Analysis
{% block css %}


{% endblock %}
{% block extra\_header %}{% endblock %}
{% block extra\_css %}{% endblock %}

{% include "navbar.html" %}
  
  
  

{% if messages %}
{% for message in messages %}

{{ message }}

{% endfor %}
{% endif %}

{% block content %}
{% endblock %}

---


© 2017 - Developed by Laboratory of Clinical Genomics - Source Code and Issues

{% block javascript %}
{% block extra\_js %} {% endblock %}
{% endblock %}
