# Supplementary material for: Mendel,MD: A user-friendly open-source web tool for analyzing WES and WGS in the diagnosis of patients with Mendelian disorders
Source: PLoS Comput Biol. 2017 Jun 8;13(6):e1005520. doi: 10.1371/journal.pcbi.1005520 (PMC5464533; doi:10.1371/journal.pcbi.1005520)
Supplement: S1 Code — Last version of the source-code of Mendel,MD. (ZIP) [file pcbi.1005520.s004.zip › mendelmd-master/mendelmd_source/templates/index.html]

{% extends "base.html" %}
{% load i18n %}
{% load account %}
{% block head\_title %}{% trans "Mendel,MD" %}{% endblock %}
{% block content %}


# Welcome to Mendel,MD!

This is an online tool created to help doctors and scientists to identify disease causing variants using exome/genome sequencing data from patients with mendelian disorders.

Sign Up » or Upload a VCF

## Filter Analysis

With this tool, you can quickly identify candidate variants of causing Mendelian disorders in your affected individuals. By excluding variants according to different criterias such as low frequency in public databases (Ex. 1000genomes, dbSNP138 and Exome Sequencing Project) and scores of patogenicity (Ex. SIFT, POLYPHEN2 and CADD) you can quickly reduce the number of variants from your initial list from thousands to a few dozens using just a few clicks.

## Family Analysis

With this tool, you can identify mendelian variants, de novo variants and compound heterozygous variants using exome data from a famillies (Ex. trios, quartets or even more individuals). This tool helps you tremendously to reduce the number of candidate variants when investigating mendelian disorders in Families.

{% endblock %}
