# Supplementary material for: Mendel,MD: A user-friendly open-source web tool for analyzing WES and WGS in the diagnosis of patients with Mendelian disorders
Source: PLoS Comput Biol. 2017 Jun 8;13(6):e1005520. doi: 10.1371/journal.pcbi.1005520 (PMC5464533; doi:10.1371/journal.pcbi.1005520)
Supplement: S1 Code — Last version of the source-code of Mendel,MD. (ZIP) [file pcbi.1005520.s004.zip › mendelmd-master/mendelmd_source/templates/navbar.html]

{% load static i18n %}

Toggle navigation


Mendel,MD

- Upload a VCF
- Dashboard
- 1-Click
- Options 
  - Individuals
  - Diseases
  - Genes
- Tools
  - 1-Click
  - Filter Analysis
  - Family Analysis
  - {% trans "Comparison" %}
- Docs
{% if request.user.is\_staff %}- {% trans "Statistics" %}
- {% trans "Databases" %}
{% endif %}
{% if user.is\_authenticated %}

- Settings 
  - Change Password
  - Change E-mail
  - Logout

{% else %}
 {% csrf\_token %}

Sign in

{% endif %}
