# Supplementary material for: Mendel,MD: A user-friendly open-source web tool for analyzing WES and WGS in the diagnosis of patients with Mendelian disorders
Source: PLoS Comput Biol. 2017 Jun 8;13(6):e1005520. doi: 10.1371/journal.pcbi.1005520 (PMC5464533; doi:10.1371/journal.pcbi.1005520)
Supplement: S1 Code — Last version of the source-code of Mendel,MD. (ZIP) [file pcbi.1005520.s004.zip › mendelmd-master/mendelmd_source/templates/pages/home.html]

{% extends "base.html" %}
{% block content %}

# Welcome to Mendel,MD!

This is an online tool that can help you identify disease causing variants using exome sequencing data from patients with mendelian disorders.

Learn more »

## Filter Analysis

With this tool you can quickly identify candidate variants of causing mendelian disorders in your affected individuals. By excluding variants according to different criterias such as low frequency in databases (1000genomes, dbSNP138 and Exome Sequencing Project) and scores of patogenicity (SIFT, POLYPHEN, CADD) you can quickly reduce the number of variants from your initial list.

Run this analysis »

## Family Analysis

With this tool you can search for mendelian variants, de novo variants and compound heterozygous variants by using the exome data from a famillies (Ex. trios, quartets and with more individuals). This helps you to reduce a lot the number of candidate variants when investigating mendelian disorders.

Run this analysis »

{% endblock content %}
