# Supplementary material for: Mendel,MD: A user-friendly open-source web tool for analyzing WES and WGS in the diagnosis of patients with Mendelian disorders
Source: PLoS Comput Biol. 2017 Jun 8;13(6):e1005520. doi: 10.1371/journal.pcbi.1005520 (PMC5464533; doi:10.1371/journal.pcbi.1005520)
Supplement: S1 Code — Last version of the source-code of Mendel,MD. (ZIP) [file pcbi.1005520.s004.zip › mendelmd-master/mendelmd_source/templates/variants.html]

{% load filter\_extras %}
{% load humanize %}

{% regroup variants by gene as gene\_list %}
{% for gene in gene\_list %}
{% if gene.grouper %}|  |  |  |  |  |  |  |  |  |  |  |  |  |  |  |  |  |  |  |  |  |  |  |  |  |  |
| --- | --- | --- | --- | --- | --- | --- | --- | --- | --- | --- | --- | --- | --- | --- | --- | --- | --- | --- | --- | --- | --- | --- | --- | --- | --- |
| {{ gene.grouper }} | | | | | | | | | | | | | | | | | | | | | | | | | |
{% endif %}| Omim - GeneCards - NCBI | | | | | | | | | | | | | | | | | | | | | | | | | |
|  | Options | Individual | Chr | RsId | Pos | Qual | Ref | Alt | Filter | Gen | Read Depth | Effect | Impact | Func Class | 1kgenomes | dbSNP | ESP6500 | Sift | PP2 | CADD | DANN | CLN | TRAIT |  |  |
{% for variant in gene.list %}|  | View | {{ variant.individual }} | {{ variant.chr }} | {{ variant.variant\_id }} {% if variant.variant\_id != "." %}   dbSNP |{% endif %} {{ variant.pos }} | {{ variant.qual }} | {{ variant.ref\|truncatechars:5 }} | {{ variant.alt\|truncatechars:5 }} | {{ variant.filter }} | {{ variant.genotype }} | {{ variant.read\_depth }} | {{ variant.snpeff\_effect }} | {{ variant.snpeff\_impact }} | {{ variant.snpeff\_func\_class }} | {{ variant.genomes1k\_maf\|floatformat:5\|intcomma }} | {{ variant.dbsnp\_maf\|floatformat:5\|intcomma }} | {{ variant.esp\_maf\|floatformat:5\|intcomma }} | {{ variant.sift\|floatformat:2 }} | {{ variant.polyphen2\|floatformat:2 }} | {{ variant.CADD\_raw\|floatformat:2 }} | {{ variant.DANN\_score\|floatformat:2 }} | {{ variant.clinvar\_clnsig }} | {{ variant.clinvar\_trait }} |  |  |
{% endfor %}
{% endfor %}
